# RAM-UNet: an improved U-Net–based semantic segmentation model for the main stem of mature soybean plants

**DOI:** 10.3389/fpls.2026.1779621

**Published:** 2026-03-10

**Authors:** Li Zhu, Wen Li, Haitao Fu, Xiaoyao Li, Yuxuan Feng

**Affiliations:** College of Information Technology, Jilin Agricultural University, Changchun, China

**Keywords:** attention mechanism, deformable convolution, mature soybean plants, multi-scale feature fusion, semantic segmentation

## Abstract

As the key structure connecting the vegetative and reproductive organs of soybean plants, the main stem plays a crucial role, and its morphological parameters serve as core phenotypic indicators for evaluating plant growth, lodging resistance, and yield potential. At the mature stage, the main stem exhibits high similarity to pods in color and texture, along with complex curvature and severe occlusion by pods and leaves, making accurate and continuous extraction challenging for conventional segmentation methods. To address this, this study proposes RAM-UNet, a high-precision semantic segmentation model based on an improved U-Net architecture. The model adopts ResNet50 as the backbone and replaces standard convolutions with deformable convolutions to capture curved stem morphology and improve feature extraction for low-contrast edges. In the encoder, the Convolutional Block Attention Module (CBAM) is combined with an improved atrous spatial pyramid pooling (ASPP) module (C-ASPP) with four dilation rates, enhancing multi-scale feature representation compared to the original three-rate design. A multi-scale attention aggregation (MSAA) module in the decoder improves continuity and integrity of stem boundaries. During training, a composite loss function combining Dice loss and cross-entropy loss is employed to mitigate foreground pixel sparsity. Experimental results on a self-constructed dataset show that RAM-UNet achieves a mean Intersection over Union (mIoU) of 90.58%, with Recall and Precision reaching 94.99% and 94.58%, respectively. Compared with U-Net, DeepLabv3+, PSPNet, and SegNet, RAM-UNet improves mIoU by 6.41%, 10.51%, 22.41%, and 17.37%, respectively. Automatically measured stem lengths show high agreement with manual measurements (R² = 0.9746), validating practical applicability. RAM-UNet also generalizes well on the public PASCAL VOC 2012 dataset, achieving an mIoU of 73.14%. The results indicate that the proposed model enables high-precision and continuous segmentation of main stems in mature soybean plants, providing an effective technical solution for automated and non-destructive measurement of crop phenotypic parameters.

## 1 Introduction

As a globally important oilseed and protein crop (Asbridge, 1995; Liu, 1997), soybean plays a critical role in enhancing yield to ensure the security of China’s food supply chain. The breeding of high-yielding and lodging-resistant cultivars relies heavily on the accurate and efficient characterization of key plant phenotypic traits. Among these traits, the morphological structure of the main stem directly reflects plant growth status, lodging resistance, and yield potential (Wilcox and Sediyama, 1981; Chen et al., 2024). Therefore, achieving accurate and automated extraction of main stem phenotypic parameters has become a fundamental and urgent requirement in modern intelligent breeding and crop management. Traditional methods relying on manual measurements are time-consuming and labor-intensive, incur high costs, and are susceptible to subjective bias, making them inadequate for large-scale phenotypic studies (Ghanem et al., 2015; Singh et al., 2016; Li et al., 2020). Computer vision-based non-contact measurement technologies provide an ideal solution to this problem, with the key challenge being the accurate segmentation of target organs from complex background images. However, the structural complexity of mature soybean plants, together with the high similarity in color and texture between the main stem, pods, and lateral branches, as well as severe mutual occlusion, poses significant challenges to achieving accurate and continuous automated segmentation.

Early studies on plant stem segmentation primarily relied on traditional digital image processing techniques. Xiushan et al. (Xiushan et al., 2015) employed adaptive algorithms to perform threshold-based image segmentation for extracting stem features of tobacco plants. Choudhury et al. (Choudhury et al., 2017) applied the Otsu thresholding method to maize stem analysis. However, the segmentation performance was highly dependent on complex preprocessing steps. Wei et al. (Wei et al., 2014) analyzed specific color factors and isolated branch regions by constraining image contrast. Xiang et al. (Xiang et al., 2022) estimated plant height by segmenting scale images in the HSV color space, but this approach was highly sensitive to illumination conditions during image acquisition, resulting in relatively large measurement errors. Xiong et al. (Xiong et al., 2018) utilized a fuzzy C-means clustering variant to delineate litchi branch regions from image data. Overall, the performance of these methods relies heavily on manually designed features. Their segmentation accuracy and robustness are markedly limited when confronted with complex scenarios involving high color similarity and severe occlusion between the main stem, pods, and lateral branches of mature soybean plants, making them inadequate for automated and high-throughput phenotypic analysis.

To address the shortcomings of conventional approaches, deep learning approaches that can automatically learn high-level features have gradually become mainstream and have demonstrated remarkable performance in the segmentation of various soybean organs. For instance, to address leaf overlap and disease in soybean, da Silva et al. (da Silva et al., 2025) developed a dual-decoder framework derived from the DeepLabv3+ architecture, enabling simultaneous semantic segmentation of soybean leaves and markers as well as pixel-area estimation, thereby providing a non-destructive solution for leaf physiological monitoring. Wu et al. (Wu et al., 2024) introduced a Mask R-CNN-based variant for accurate pixel-level extraction of densely aggregated soybean rust spores. In addressing low contrast and class imbalance, soybean root segmentation serves as a classic scenario. Wang et al. (Wang et al., 2019) employed a SegNet encoder-decoder architecture to achieve automatic segmentation and length estimation of soybean roots in soil backgrounds, while Xu et al. (Xu et al., 2022) enhanced the U-Net framework by introducing a dual-attention design, significantly improving the segmentation accuracy of hydroponic soybean seedling roots. In addressing multi-scale and densely occluded targets, soybean pod segmentation studies have provided valuable insights. Yang et al. (Yang et al., 2022) developed a Mask R-CNN approach using a synthetically generated dataset, enabling high-throughput segmentation and morphological measurement of detached soybean pods, effectively mitigating the challenge of limited annotated data. Yang et al. (Chen et al., 2024) improved the Mask Transfiner network to develop the RefinePod model. By incorporating a dual attention mechanism and a center loss function, the model enables segmentation of soybean pods and fine-grained classification of seed numbers, advancing pod phenotypic analysis to a more detailed level. Taken together, existing research highlights the strong capability of deep learning approaches in addressing challenging soybean segmentation problems, providing a strong foundation for the automated and high-precision segmentation of the main stem addressed in the present work.

Despite these advances, automated fine-grained segmentation of the main stem remains challenging. As a key structure connecting the vegetative and reproductive organs, the stem, particularly the main stem, still faces a series of challenges in automated fine-grained segmentation, and related research remains at an exploratory and developmental stage (Kato et al., 2015; Liu et al., 2016). For detached samples, Li et al. (Li et al., 2022) proposed the SPM-IS method based on the YOLACT instance segmentation framework using ResNet-101-FPN as the feature extraction network and integrates a multi-task loss function for optimization. In laboratory conditions, the method was tested on detached soybean stems, achieving a mean Average Precision (mAP) of 93.5% for stem segmentation. However, this approach relies on manually disassembling plants and imaging them individually, effectively avoiding occlusion issues inherent to complex plant structures. Jiajun et al. (Jiajun et al., 2024) employed U²-Net to address occlusion caused by densely clustered pods on soybean stems. Using its multi-scale feature extraction structure, for stem region segmentation, the proposed approach achieved an mIoU score of 82.6%, providing accurate pixel-level inputs for subsequent stem diameter estimation. Other studies have applied improved U-Net and DeepLab-based architectures to achieve high-precision stem or branch segmentation in various plant species. Yan et al. (Yan et al., 2021) used a modified U-Net to perform high-precision segmentation of wheat stem microscopic cross-sections. Zhendong et al. (Zhendong et al., 2024) developed a refined U-Net architecture aimed at cherry branch diameter estimation by replacing the backbone with VGG16 and incorporating a spatial attention module (SAM), achieving an mIoU of 77.9% in dormant cherry branch segmentation. Yi et al. (Yi et al., 2023) developed the AC-UNet model for semantic segmentation of stems and leaves in Betula luminifera, achieving an mIoU of 87.5% by modifying the backbone and improving the U-Net architecture. In the segmentation of litchi stems, Xie et al. (Xie et al., 2022) and Peng et al. (Peng et al., 2020) both based their methods on the DeepLabV3+ architecture, incorporating improvements such as backbone replacement and integration of attention modules, which effectively enhanced segmentation accuracy. Zhengkai et al. (Zhengkai et al., 2023) introduced CBAM and modified the loss function in PSP-Net to extract winter kiwifruit branch regions, improving branch continuity and segmentation accuracy. Collectively, although these studies cover multiple plant species, automated fine-grained segmentation of soybean stems, particularly the main stem during the critical period of yield formation at maturity, remains insufficiently explored. The structural complexity of mature soybean plants, together with the overlap and occlusion of the main stem by pods and lateral branches that are similar in color and texture, results in blurred target boundaries and feature confusion. Consequently, existing segmentation models struggle to achieve accurate and continuous main stem extraction, which in turn limits the precision and reliability of subsequent plant phenotypic measurements.

The classical U-Net model, with its encoder–decoder architecture and skip connections, possesses inherent advantages in integrating multi-scale contextual information and restoring fine structures of target objects, making it particularly suitable for addressing boundary blurring caused by occlusion. Therefore, in this study, U-Net was selected as the foundational framework to develop an improved RAM-UNet model aimed at achieving precise semantic segmentation of the main stem in mature soybean plants. Specifically, the main contributions of this study are summarized:

1. ResNet50 replaced the original backbone network, while traditional convolutional operations in the encoding stage were exchanged for deformable convolutional layers to enable adaptive modeling of the curved morphology of soybean stems.
2. The encoder is augmented with a C-ASPP module at its final stage, integrating CBAM attention and employing a combination of dilation rates (1, 2, 7, 15) to enhance multi-scale contextual perception.
3. A multi-scale attention aggregation (MSAA) module was innovatively integrated into the decoder to construct an efficient feature refinement pathway, effectively addressing stem feature fusion and continuity restoration under complex occlusion.
4. To alleviate class imbalance caused by sparse foreground pixels, a loss formulation integrating Dice loss with cross-entropy loss was employed, and the segmentation results were further used to achieve automated and accurate measurement of main stem length.

## 2 Materials and methods

### 2.1 Dataset establishment for mature soybean plants

#### 2.1.1 Data collection

**Figure 1:**
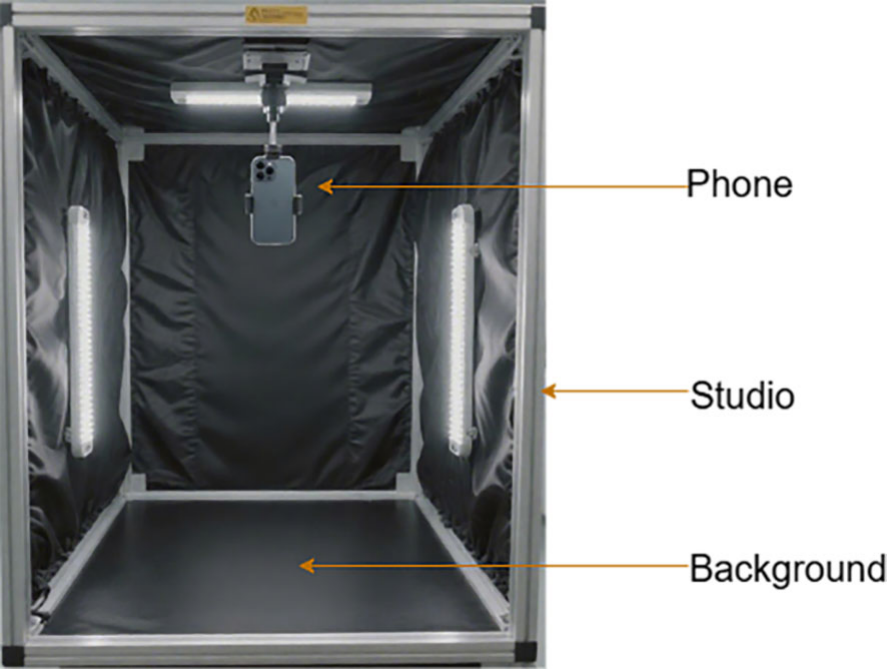
Imaging setup for collecting mature soybean plants.

Representative Northeast Yellow Soybean, widely cultivated in China’s major soybean-producing regions, was selected as the experimental sample. All samples were grown in a standardized experimental field at the Jilin Agricultural University Research and Demonstration Base in Changchun, Jilin Province (geographic coordinates: 43.82°N, 125.41°E), and were collected after the plants reached physiological maturity (R8 stage). The collection process was completed between October 10 and 20, 2023. To maintain the freshness of the samples and ensure consistent imaging conditions, a batch of plants was collected daily from different areas of the field between 10:00 AM and 3:00 PM, immediately transferred to a fixed indoor imaging platform, and standardized imaging of all samples in the batch was quickly completed. To acquire high-quality images, data were captured in a controlled indoor environment using a pure black background to minimize interference and LED lights with stable color temperature to provide uniform illumination and suppress shadows. The images were captured using a smartphone (iPhone 14 Pro Max), positioned 80 cm directly above the samples, with the camera axis perpendicular to the plant surface. This setup allowed for the capture of the plants’ morphological and occlusion diversity from standardized perspectives. Each plant was subjected to planar rotation and local posture adjustments, resulting in four images from different angles. Based on this approach, a dataset focused on the segmentation of mature soybean stems was constructed, consisting of 1,400 raw images from 350 plants, each with a resolution of 3024×4032 pixels in JPG format. To establish the pixel-to-physical size conversion, a standard ruler was placed within the field of view. The data acquisition setup is illustrated in **Figure 1**.

#### 2.1.2 Data preprocessing

**Figure 2:**
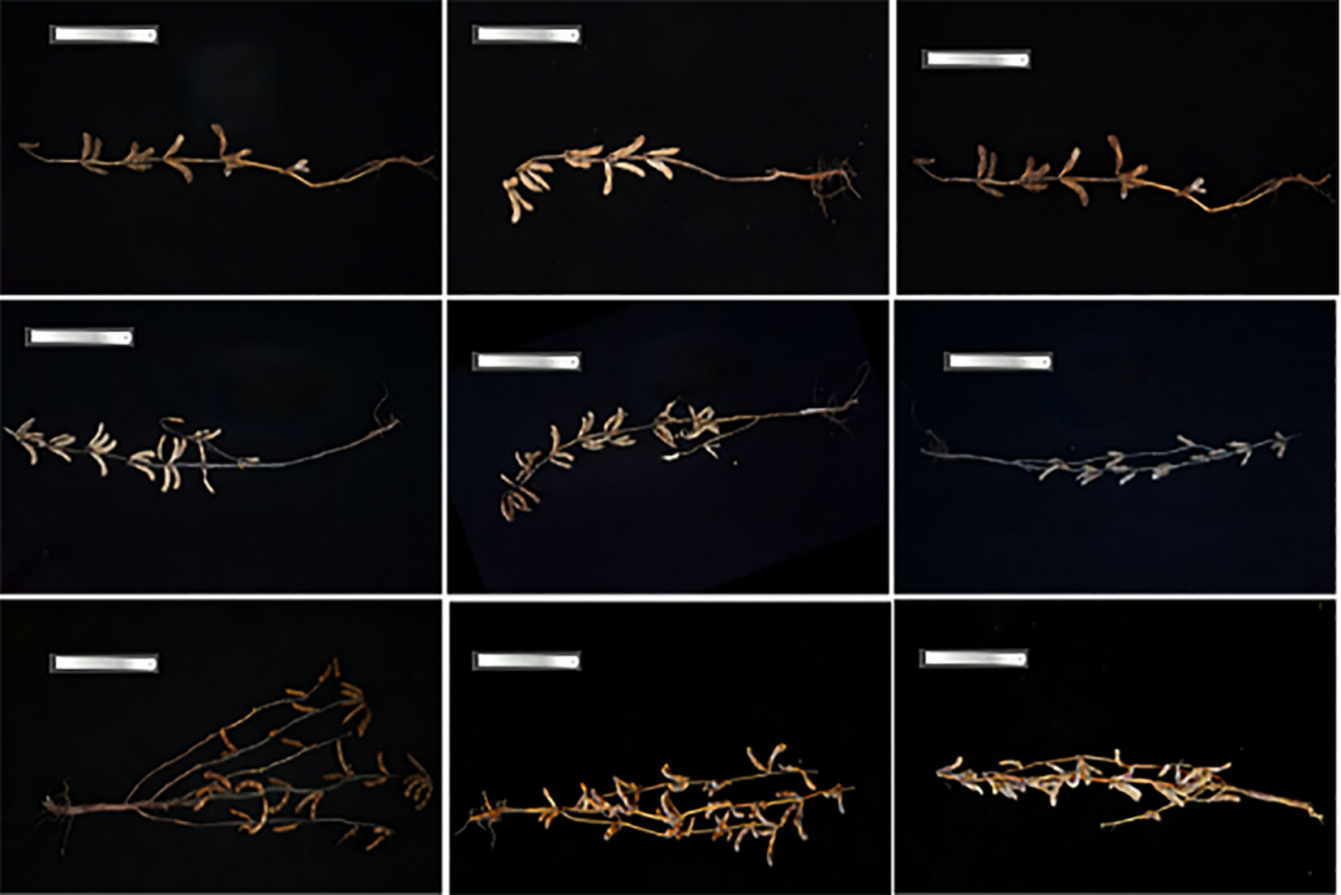
Images of mature soybean plants.

Examples of the dataset are shown in **Figure 2**. The mature soybean plant samples in the dataset exhibit rich morphological diversity. In terms of plant architecture, the dataset includes single-branch, double-branch, and complex-branch types. Regarding the main stem morphology, it covers a wide range of postures from nearly upright to prominently curved, with substantial variation in length and thickness from base to apex, enhancing the model’s generalization ability across different growth forms. To obtain ground truth labels for model training, the images were manually annotated at the pixel level using the LabelMe tool (Russell et al., 2008). A Python script was then developed to automatically parse the JSON annotation files generated by LabelMe and convert them into binary label images.

**Figure 3:**
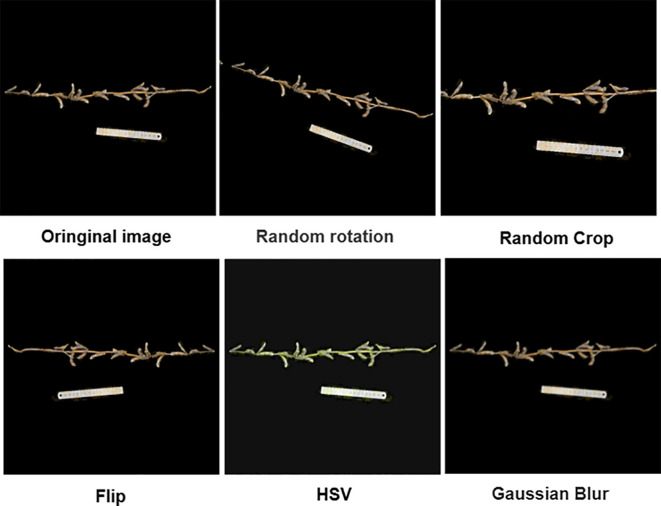
Examples of data augmentation.

To scientifically assess the model’s generalization performance on different stem morphologies and ensure the fairness and statistical validity of the evaluation, this study adopted a strict plant-based partitioning strategy. To ensure that both the training and validation sets adequately represent a range of stem morphologies, from simple to complex, and to avoid evaluation bias due to skewed data distribution, all 350 plants were manually classified into three categories based on stem morphology complexity: simple, double, and complex branching. Within each category, plants were used as the basic sampling unit, and a stratified random sampling strategy was employed, with a fixed random seed of 11 to ensure reproducibility. The plants were randomly assigned to the training and validation sets in an 8:2 ratio. The key principle of this strategy is that all images from the same plant are assigned exclusively to one set, ensuring complete independence of the training and validation sets at the biological sample level and fundamentally eliminating data leakage. As a result, 280 plants were assigned to the training set, and 70 plants to the validation set. Multiple augmentation techniques were randomly applied, including rotation, random cropping, horizontal flipping, HSV color space perturbation, and Gaussian noise addition. Examples of the augmented images are shown in **Figure 3**. Finally, all images and their corresponding label files were organized and converted into Pascal VOC dataset format to facilitate subsequent model training and evaluation.

#### 2.1.3 Acquisition of main stem length data

The main stem length of soybean plants is defined as the straight-line distance from the root-stem junction to the apex of the plant along the main stem growth direction (Yang et al., 2021). In this study, the conventional manual measurement method was applied to morphologically intact mature soybean plants. Each plant was laid flat on a measurement platform, and the root-stem junction was identified as the starting point. A measuring tool was then aligned with the natural growth trajectory of the main stem to record the distance to the stem apex. To improve measurement reliability, each plant was measured three times, and the mean value was taken as the final main stem length. All measurement data were carefully recorded and retained for subsequent analysis.

### 2.2 U-Net model architecture

**Figure 4:**
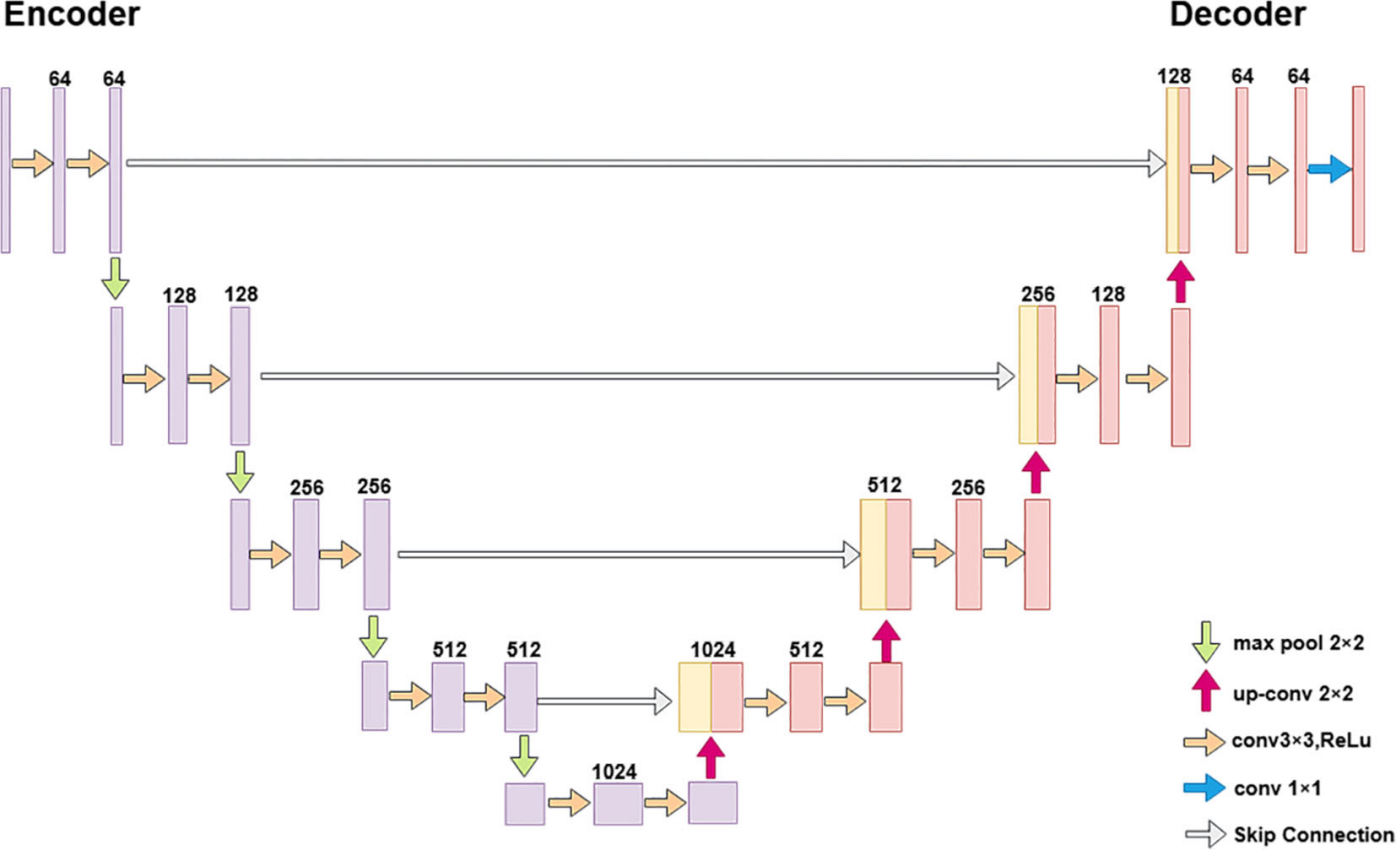
Original U-Net model architecture.

U-Net was first introduced in the work of Ronneberger et al (Ronneberger et al., 2015). and has since shown broad applicability across a variety of image analysis tasks, such as crop phenotyping and the identification of pests and diseases. The model adopts a classical U-shaped encoder–decoder architecture, as illustrated in **Figure 4**. In the encoder path, feature extraction is performed through four repeated blocks of 3×3 convolutions, ReLU activations, and 2×2 max pooling operations, progressively capturing deep semantic information. In the decoder path, 2×2 transposed convolutions are employed for upsampling, and skip connections concatenate high-resolution features from the corresponding encoder layers with the decoder features. This enables the network to recover spatial resolution while simultaneously integrating multi-scale contextual information. Finally, a 1×1 convolution outputs a segmentation map of the same size as the input. Due to its simplicity, extensibility, and ability to achieve precise pixel-level predictions with limited training data, U-Net has become a widely used baseline framework in semantic segmentation tasks.

### 2.3 Improved U-Net semantic segmentation model

**Figure 5:**
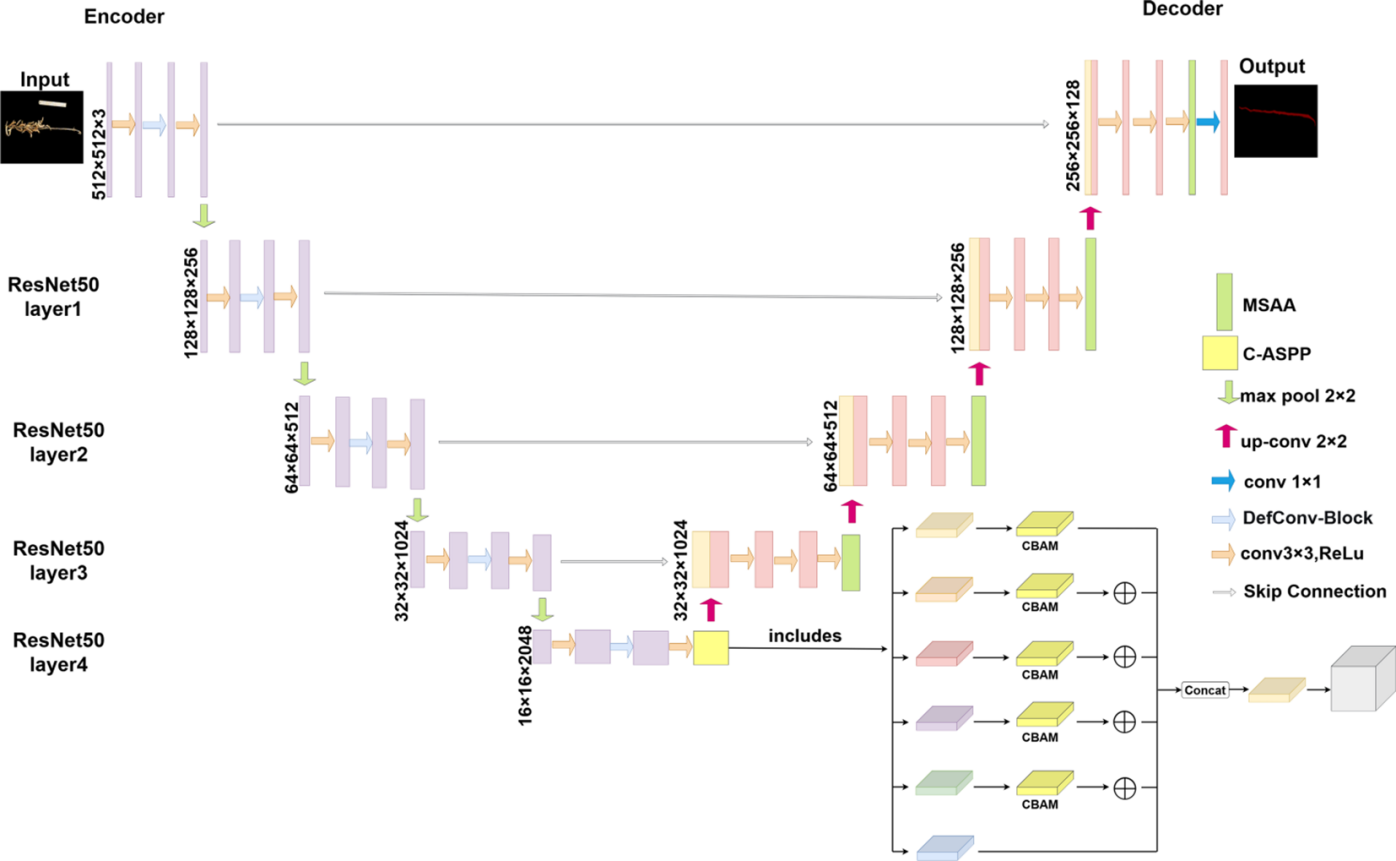
Architecture of the improved RAM-UNet model.

The network has been widely adopted for segmentation tasks. However, its basic structure exhibits certain limitations when applied to the main stem of mature soybean plants. The encoder employs standard convolutions, which are insufficiently flexible to adapt to the highly variable curvature of the main stem and have limited capability in extracting features from low-contrast edges. Additionally, the network lacks an effective multi-scale contextual awareness mechanism at higher levels, making it difficult to distinguish the main stem from pods with highly similar color and texture. In the decoder, the simple skip connections and feature concatenation operations are inefficient in integrating deep semantic features with shallow details under severe occlusion, often resulting in fragmented segmentation and poor detail recovery. To address these issues, the present study takes U-Net as the baseline and enhances it at three levels—feature extraction, contextual awareness, and feature fusion—resulting in the proposed RAM-UNet model. **Figure 5** illustrates the architecture of the proposed network.

In the encoder, a ResNet50 backbone is employed to strengthen feature extraction. To accommodate the curved morphology of stems, the standard 3×3 convolutions within the Bottleneck modules of the backbone were replaced with deformable convolutions, enabling the network to dynamically adjust its receptive field and more accurately capture nonlinear edges. To further enhance multi-scale contextual information, a C-ASPP module is introduced at the encoder output. This module adopts dilation rates of 1, 2, 7, and 15 tailored to stem-scale characteristics, with CBAM applied for feature refinement. Such a design adaptively fuses cross-scale features ranging from local details to long-range structures, effectively improving the model’s discriminative ability between visually similar organs and its semantic understanding of occluded regions.

In the decoder, a Multi-Scale Attention Aggregation (MSAA) module was introduced to improve feature fusion and detail restoration. This module adaptively weights and fuses shallow, high-resolution features from the encoder with deep, semantic features from the decoder through parallel multi-scale convolutions and a dual attention mechanism, thereby enhancing the selection and integration of critical details. Subsequently, a progressive fusion strategy was employed to gradually merge the refined features with higher-resolution shallow features. After each fusion step, a 3×3 convolution was applied for smoothing, and the segmentation output was restored to the input resolution via bilinear upsampling. This design substantially improves the continuity restoration of the main stem in occluded regions while maintaining clear and precise segmentation boundaries.

### 2.4 Backbone network

**Figure 6:**
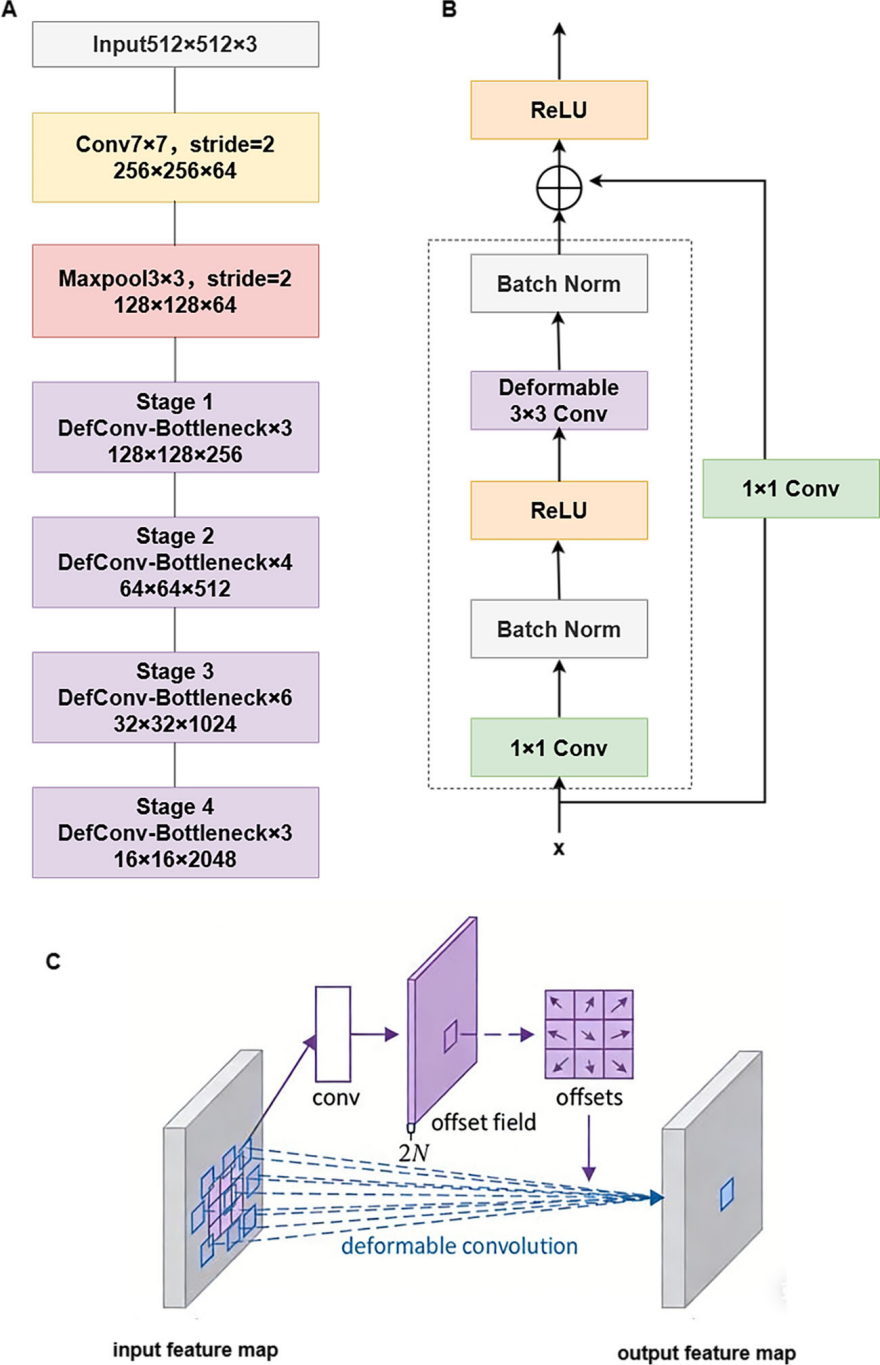
Design of the ResNet50-DCN backbone network. **(A)** Backbone network selection. **(B)** Structure of the deformable residual module. **(C)** Schematic illustration of the deformable convolution principle.

In the classical U-Net architecture, the encoder is typically composed of simple stacked convolutions, whose fixed receptive fields struggle to adapt to the irregular shapes of mature soybean main stems, which are often curved and exhibit blurred edges. This limitation reduces the network’s ability to capture critical stem contours, subsequently affecting the accuracy and continuity of segmentation. To achieve adaptive feature extraction for stem morphology while maintaining strong representational capacity, ResNet50 was selected as the backbone for the improved model. Leveraging its residual learning mechanism and deep hierarchical structure, ResNet50 can extract robust, multi-level semantic features, providing a solid foundation for fine-grained segmentation. However, to better accommodate the irregular geometry of soybean stems, key modifications were made to the core convolutional operations, as shown in **Figure 6**. The main contribution of this work is the replacement of the standard 3×3 convolution in ResNet50’s Bottleneck module with deformable convolution. The key advancement of deformable convolution is its ability to replace the conventional fixed sampling grid with dynamically learned offsets, enabling adaptive feature extraction. Parallel branches are employed to learn spatial offsets for each sampling point, allowing the convolutional kernel to adjust its receptive field distribution according to local feature content. During feature sampling, the network uses these learned offsets to perform adaptive resampling of the feature maps, generating representations that better conform to the true morphology of the target. This design substantially enhances the modeling capability for curved stem edges with minimal additional parameters. Deformable convolutions are integrated into the residual Bottleneck modules of ResNet50, forming deformable residual modules. This module retains the original three-layer residual design: a 1×1 convolution first adjusts the channel dimension, followed by a 3×3 deformable convolution for adaptive spatial feature extraction, and finally a 1×1 convolution restores the channel dimension. The output is added to the input via a skip connection. By maintaining the advantages of residual learning, this design allows the network to modulate its receptive field dynamically, so that convolutions can sample along the curved paths of stems. In the forward pass, the input image sequentially undergoes initial convolutional downsampling and four stages of feature extraction. Each stage contains multiple deformable residual modules, progressively deepening the network and expanding feature channels, ultimately producing high-level semantic features for subsequent decoding.

### 2.5 C-ASPP module

#### 2.5.1 ASPP

**Figure 7:**
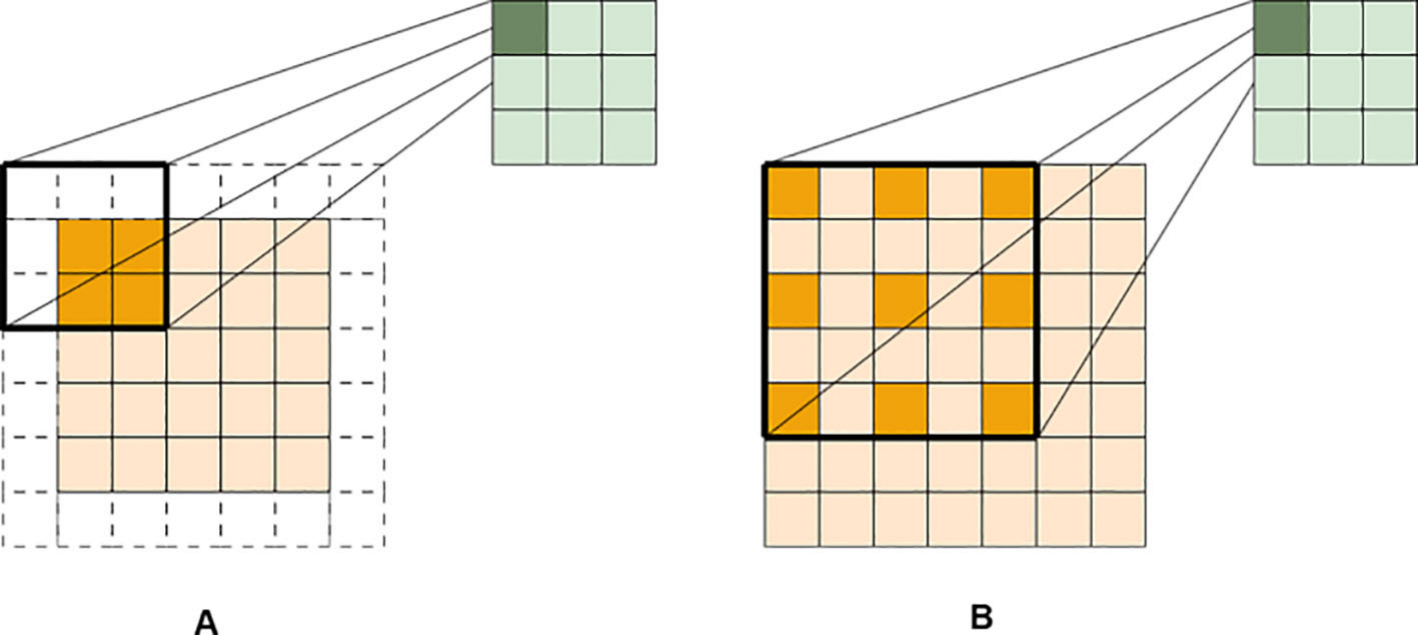
Illustration of standard and dilated convolution principles: **(A)** standard operation, **(B)** atrous operation.

ASPP (Chen et al., 2018) is a classical structure in semantic segmentation for capturing multi-scale contextual information. Its core principle lies in using a set of parallel atrous convolution layers to simultaneously capture multi-scale features without downsampling or increasing the number of parameters, a comparison with standard convolution is illustrated in **Figure 7**. For a k×k standard convolution kernel, introducing a dilation rate r results in an effective receptive field $k_{d}$ calculated as Equation 1:

(1) $$\begin{array}{c} k_{d}=k+(k-1)\times (r-1) \end{array}$$

**Figure 8:**
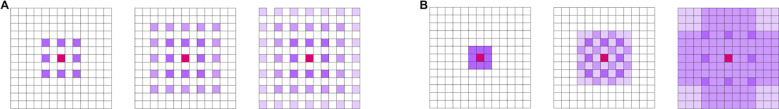
Atrous convolution configurations: **(A)** grid effect, **(B)** our optimized design.

The standard ASPP module typically consists of five parallel branches: a 1×1 convolution, three 3×3 atrous convolutions with dilation rates of 6, 12, and 18, and a global average pooling layer. The outputs of all branches are upsampled and concatenated for feature fusion. However, when excessively large dilation rates (e.g., 12 and 18) are stacked, the effective receptive field of pixels in high-level feature maps exhibits a sparse grid pattern, leading to blind spots in perceiving continuous stem structures, known as the “gridding effect”, as illustrated in **Figure 8**. Moreover, the default dilation rate combination (6, 12, 18) is designed for general scenarios, and its physical receptive field span does not match the organ-level scales of soybean plants, resulting in coarse feature extraction and limited ability to capture fine details. To fundamentally address the aforementioned issues, the ASPP module was structurally optimized and its parameters reconfigured in this study. First, in terms of branch design, a standard 3×3 convolution branch with a dilation rate of r=1 was added to the three existing atrous convolution branches in the standard ASPP, forming a multi-scale feature extractor with a more continuous receptive spectrum and enhanced sensitivity to local details. The r=1 branch focuses on fundamental local feature interactions and edge responses, collectively providing seamless coverage from pixel-level to global context. Second, in terms of parameter configuration, guided by the mathematical constraints for avoiding grid artifacts and considering the biophysical scale of soybean plants, the four atrous convolution branches were assigned dilation rates of 1, 2, 7, and 15, respectively. To prevent the grid effect, a sequence of consecutive dilation rates must satisfy the “no common factor greater than 1” criterion (Wang et al., 2018), which can be formalized as Equation 2:

(2) $$\begin{array}{c} gcd(r_{1},r_{2},\cdots ,r_{n})=1 \end{array}$$

Here, gcd denotes the greatest common divisor. Based on this principle, the dilation rates were set to 1, 2, 7, and 15, forming a gradient combination that covers a range of receptive fields, thereby ensuring dense and continuous coverage of the target region and fundamentally eliminating the grid effect. Smaller dilation rates (e.g., 1 and 2) focus on fine local features such as stem edge textures; a medium dilation rate (7) captures the connectivity between lateral branches and the main stem; and the largest dilation rate (15) integrates global context, encompassing the overall plant morphology and spatial layout. This design enables the model to achieve comprehensive and adaptive feature perception of the main stem across multiple scales, from pixel-level details to organ-level structures.

#### 2.5.2 CBAM

**Figure 9:**
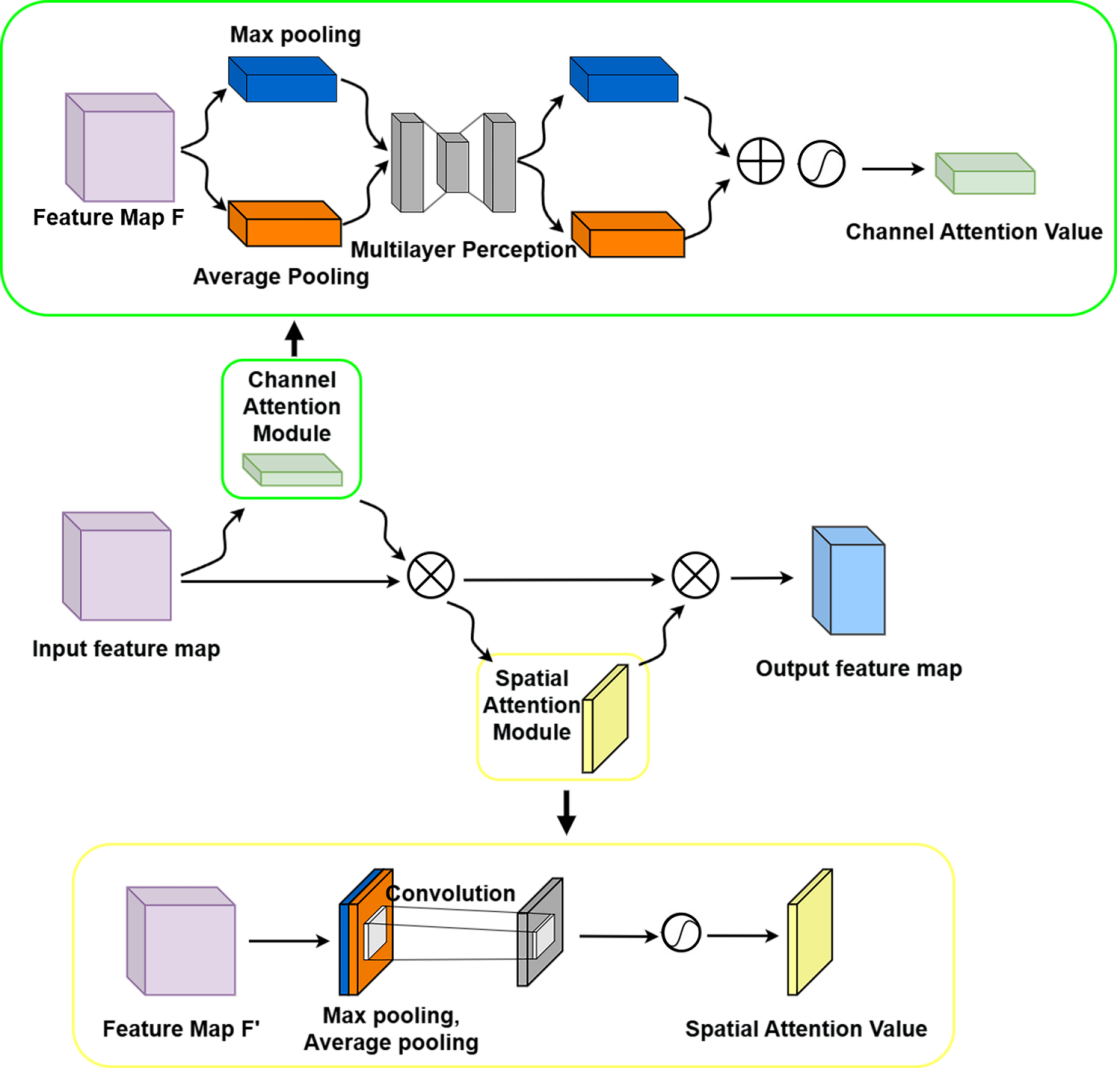
Convolutional block attention module.

To overcome the limitations of simple feature fusion in the standard ASPP module and to enable adaptive selection and enhancement of multi-scale features, the CBAM attention mechanism (Woo et al., 2018) was integrated following the ASPP module in this study. This module sequentially applies channel and spatial attention submodules to adaptively recalibrate the feature map along both the channel and spatial dimensions, allowing the network to focus on key features while suppressing irrelevant information. The structure is illustrated in **Figure 9**. By performing dual dynamic calibration to enhance feature discriminability, the input feature map F is processed through global max pooling and global average pooling. These are fed into a shared-weight multilayer perceptron (MLP) to generate the channel attention map ${\text{M}}_{\text{c}}(\text{F})$ which is then activated by a Sigmoid function and multiplied element-wise with F to produce the channel-weighted feature map ${\text{F}}^{'}$. The computation is formulated as follows Equation 3:

(3) $$\begin{array}{c} M_{c}(F)=\mathrm{\&x03C3;}[M_{lp}(F_{avg})+M_{lp}(F_{\max})] \end{array}$$

${\text{M}}_{\text{c}}(\text{F})$ denotes the channel attention weights, where σ denotes the Sigmoid activation function, and ${\text{M}}_{\text{lp}}$ denotes a shared-weight multilayer perceptron. ${\text{F}}_{\text{avg}}$ and ${\text{F}}_{\text{max}}$ are the spatial feature maps obtained via average and max pooling, respectively. The feature map ${\text{F}}^{'}$ is subjected to the same dual-pooling operations, and the results are concatenated and passed through a convolutional layer to generate the spatial attention map ${\text{M}}_{s}(\text{F"})$. After Sigmoid activation, the final feature map ${\text{F}}^{'}$ is obtained through element-wise multiplication of ${\text{M}}_{\text{s}}(\text{F'})$ and F', enabling adaptive weighting across both channel and spatial dimensions. The computation is formulated as follows Equation 4:

(4) $$\begin{array}{c} M_{s}(F^{'})=\mathrm{\&x03C3;}(f^{7\times 7}[{F^{'}}_{avg};{F^{'}}_{\max}]) \end{array}$$

In the equation, $M_{S}(F^{'})$ denotes the spatial attention output weights, ${\text{f}}^{7\times 7}$ represents a 7×7 convolutional filter, ${{\text{F}}^{'}}_{\text{avg}}$ is the feature map obtained by channel-wise average pooling, and ${{\text{F}}^{'}}_{\text{max}}$ is the feature map obtained by channel-wise max pooling. The input feature map F is first multiplied element-wise with the channel attention module, and the resulting feature map is subsequently multiplied element-wise with the spatial attention module. After processing by CBAM, the final feature map F' is obtained. The computation can be expressed as Equation 5:

(5) $$\begin{array}{l} F^{'}=M_{c}(F)⊗F\\ F^{'}=M_{S}(F^{'})⊗F^{'} \end{array}$$

F denotes the input feature map, $F^{'}$ represents the feature map after channel attention weighting, $F^{'}$ represents the feature map after spatial attention weighting, and ⊗ denotes element-wise multiplication.

#### 2.5.3 Improved C-ASPP module

**Figure 10:**
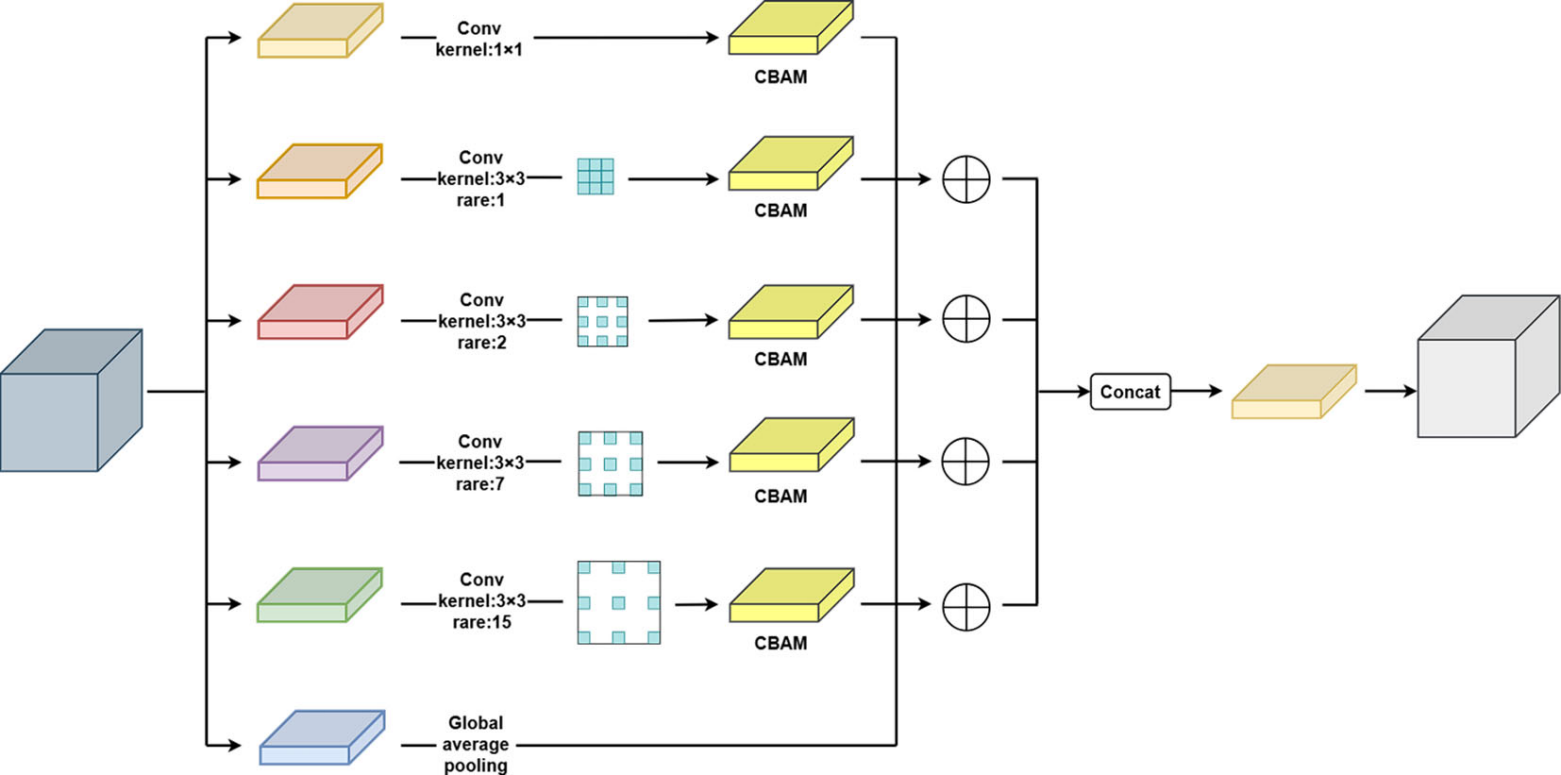
Improved C-ASPP Module Architecture.

To further enhance feature selectivity and discriminability while preserving multi-scale contextual perception, this study integrates CBAM into each feature extraction branch of the ASPP module. First, the multi-scale convolutional branches of the standard ASPP were restructured and re-parameterized. The multi-scale convolutional branches of the ASPP module were restructured and parameter-optimized. Specifically, the standard ASPP was extended by introducing an additional 3×3 convolutional branch with a dilation rate of r = 1, in addition to its original three atrous convolution branches. The dilation rates were optimized and set to 1, 2, 7, and 15 to better match the biophysical scales of soybean plants, ranging from fine local details to global structural patterns. Subsequently, a CBAM module was appended to the output of each ASPP branch to achieve adaptive feature enhancement along both the channel and spatial dimensions. Channel attention assigns adaptive weighting coefficients to different feature channels. Meanwhile, the spatial attention mechanism focuses on informative regions in the two-dimensional space. This attention-enhanced strategy enables the network to dynamically suppress background noise and reinforce critical details during multi-scale feature extraction, thereby markedly improving modeling accuracy and robustness under complex morphological conditions. **Figure 10** presents the structural design of the improved C-ASPP module.

### 2.6 MSAA module

**Figure 11:**
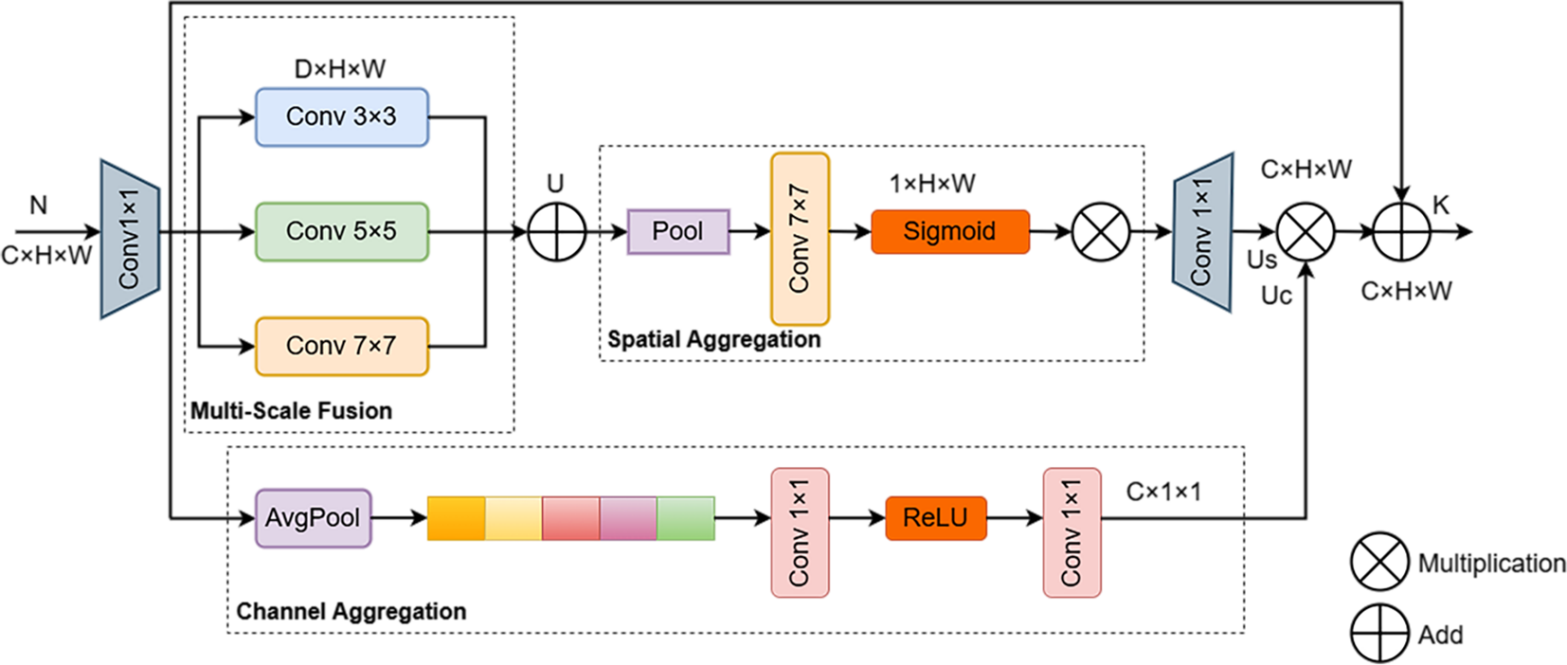
Architecture of the MSAA module.

In the decoder path of U-Net, skip connections restore target boundary details by fusing shallow, high-resolution features from the encoder with deep semantic features from the decoder. However, in images of mature soybean plants, the main stem is often severely occluded by pods and leaves, causing the skip-connection features to contain both valid edge information from fragmented stems and a substantial amount of background noise. Direct feature concatenation under such conditions tends to overwhelm critical stem-related information, thereby degrading the continuity of main stem segmentation. To effectively enhance the skip-connection features prior to feature fusion, this study introduces a Multi-Scale Attention Aggregation (MSAA) module (Liu et al., 2024), which is embedded into the decoder after the upsampling operation and before feature concatenation. The design of the MSAA module is inspired by widely used channel and spatial attention mechanisms in computer vision and aims to adaptively recalibrate feature responses. The core architecture of the MSAA module is illustrated in **Figure 11**.

Given an input feature map $\text{N}\epsilon {\text{R}}^{C\times H\times W}$, feature dimensionality reduction and multi-scale context aggregation are first performed. A 1×1 convolution layer is applied to reduce the channel dimension from C to D, thereby decreasing computational complexity Equation 6:

(6) $$\begin{array}{c} Z=Conv_{1\times 1}(N) \end{array}$$

where $ZЄ{\text{R}}^{D\times H\times W}$ denotes the dimension-reduced feature map and D represents the number of channels after dimensionality reduction. Subsequently, to capture contextual information under different receptive fields, parallel convolutions at three spatial scales (3×3, 5×5, and 7×7) are applied to Z, and their outputs are aggregated by element-wise summation Equation 7:

(7) $$\begin{array}{c} U=Conv_{3\times 3}(Z)+Conv_{5\times 5}(Z)+Conv_{7\times 7}(Z) \end{array}$$

where $\text{U}Є{\text{R}}^{\text{D}\times \text{H}\times \text{W}}$ denotes the aggregated multi-scale feature map. Based on this representation, the module recalibrates features through two parallel attention pathways. The spatial attention mechanism emphasizes salient spatial locations and modulates the reduced feature representation Z Equation 8:

(8) $$\begin{array}{c} U_{s}=W_{s}⊙Z \end{array}$$

where ${\text{U}}_{\text{s}}Є{\text{R}}^{\text{D}\times \text{H}\times \text{W}}$ denotes the spatially enhanced feature map, ${\text{W}}_{\text{s}}Є{\text{R}}^{1\times \text{H}\times \text{W}}$ denotes the spatial attention weight map, and ⊙ represents element-wise multiplication. The channel attention branch captures inter-channel dependencies and generates channel-wise attention weights to recalibrate the feature map Z Equation 9:

(9) $$\begin{array}{c} U_{c}=W_{c}⊙Z \end{array}$$

where ${\text{U}}_{\text{c}}Є{\text{R}}^{\text{D}\times \text{H}\times \text{W}}$ denotes the channel-enhanced feature map and ${\text{W}}_{\text{c}}Є{\text{R}}^{\text{D}\times 1\times 1}$ denotes the channel attention weight vector. Finally, attention interaction and residual output are performed to jointly exploit spatial and channel-wise attention cues. The spatially enhanced feature ${\text{U}}_{\text{s}}$ and the channel-enhanced feature ${\text{U}}_{\text{c}}$ are combined via element-wise multiplication to obtain an interaction feature. Subsequently, a 1×1 convolutional layer is applied to restore the channel dimension to match that of the input feature map N. A skip connection is then introduced to form a residual structure, yielding the final enhanced output feature Equation 10:

(10) $$\begin{array}{c} K=Conv_{1\times 1}(U_{s}⊙U_{c})+N \end{array}$$

where $\text{K}Є{\text{R}}^{\text{C}\times \text{H}\times \text{W}}$ represents the feature map produced by the MSAA operation. By introducing the MSAA module, the model is able to adaptively enhance key channels and spatial locations associated with the soybean main stem within the skip-connection features prior to feature fusion, while effectively suppressing responses from irrelevant background regions. This explicit feature re-calibration mechanism significantly improves the robustness of the model in recovering continuous main stem contours under severe occlusion, thereby enhancing segmentation reliability in complex plant scenes.

### 2.7 Loss function

In image segmentation tasks, the cross-entropy loss is widely used to quantify the pixel-wise discrepancy between model predictions and ground-truth labels. However, in the task of main stem segmentation for mature soybean plants, the slender stem occupies only a small proportion of the image, resulting in a severe class imbalance between foreground and background pixels. Under such conditions, the conventional cross-entropy loss is dominated by the background class, which weakens the model’s ability to learn discriminative features for sparse foreground regions and particularly degrades segmentation accuracy along object boundaries. To alleviate the class imbalance problem, this study introduces the Dice loss, which directly optimizes the overlap between the predicted mask and the ground truth by maximizing the Dice coefficient. By emphasizing region-level geometric consistency, Dice loss effectively enhances the model’s sensitivity to sparse foreground pixels. To jointly account for pixel-level classification accuracy and region-level structural similarity, a composite loss function is ultimately adopted for model training, defined as a linear combination of the Dice loss and the cross-entropy loss. The mathematical formulations are given as follows Equation 11, 12:

(11) $$\begin{array}{c} L_{CE}\;=\;-\;\frac{1}{N}\sum_{i\;=\;1}^{N}\left([,\begin{array}{c} g_{i}logp_{i}\;+\\ \left(1\;-\;g_{i}\right)\log\left(1\;-\;p_{i}\right) \end{array},]\right) \end{array}$$

(12) $$L_{\text{Dice}}=1-\frac{2\sum_{i}p_{i}g_{i}+\epsilon }{\sum_{i}p_{i}+\sum_{i}g_{i}+\epsilon }$$

where ${\text{L}}_{\text{CE}}$ denotes the cross-entropy loss, ${\text{L}}_{\text{Dice}}$ denotes the Dice loss, N refers to the total pixel count of the image, ${\text{g}}_{\text{i}}$ and ${\text{p}}_{\text{i}}$ denote the true annotation and the predicted foreground probability for pixel i, respectively, and ϵ is a smoothing term introduced to prevent division by zero. The final composite loss function ${\text{L}}_{\text{CD}}$ is defined as the weighted sum of the two losses Equation 13:

(13) $$\begin{array}{c} L_{CD}=\alpha L_{Dice}+(1-\alpha )L_{CE} \end{array}$$

where α is the weighting coefficient, with αϵ[0,1]. In this formulation, the Dice loss encourages the model to learn the global shape and continuity of the soybean main stem, whereas the cross-entropy loss provides stable pixel-wise gradients, ensuring reliable convergence during the early stages of training. The combination of the two losses enables the model to effectively handle severe class imbalance while achieving more accurate boundary delineation.

### 2.8 Main stem length measurement method

**Figure 12:**
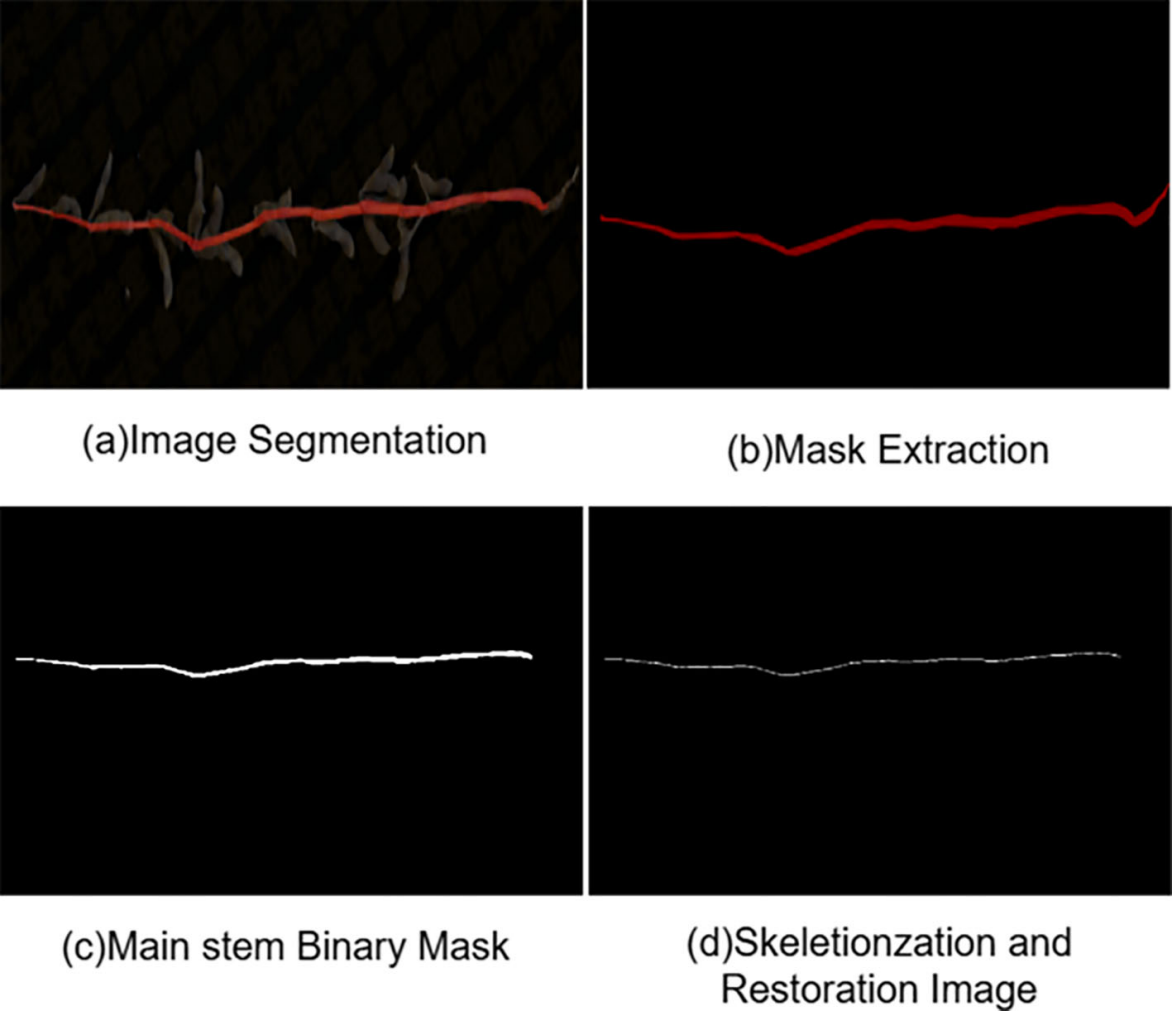
Example of main stem pixel length extraction.

To enable automated measurement of main stem length, this study establishes a complete computational pipeline that converts semantic segmentation results into physical length measurements. Starting from the semantic segmentation mask produced by the proposed RAM-UNet model, a series of standard image processing and geometric computation procedures are sequentially applied, including scale calibration, main stem skeleton extraction, and path length calculation. The key steps of the proposed pipeline are illustrated in **Figure 12**.

Scale calibration is performed using a reference ruler placed in the image. The maximum pixel distance of the ruler, denoted as ${\text{RL}}_{\text{pixel}}$, is measured and combined with its known real-world length ${\text{RL}}_{\text{real}}$ to derive the pixel-to-length conversion factor r, which establishes the correspondence between image pixels and physical distance Equation 14:

(14) $$\begin{array}{c} r=\frac{RL_{real}}{RL_{pixel}} \end{array}$$

The segmented binary mask of the main stem is then subjected to morphological thinning to extract a single-pixel-wide centerline. This skeleton is represented as an ordered set of pixel coordinates ${\left\{({\text{x}}_{\text{i}},{\text{y}}_{\text{i}})\right\}}_{\text{i}=1}^{\text{n}}$. The pixel-wise path length of the main stem, denoted as ${\text{L}}_{\text{pixel}}$, is subsequently calculated by summing the Euclidean distances between consecutive points along the skeleton Equation 15:

(15) $$L_{\text{pixel}}=(\sum_{i=1}^{n-1}\sqrt{{\left(x_{i+1}-x_{i}\right)}^{2}+{\left(y_{i+1}-y_{i}\right)}^{2}})$$

Finally, the pixel length is mapped to the actual physical length of the main stem, ${\text{L}}_{\text{real}}$, using the conversion factor r derived from the scale calibration Equation 16:

(16) $$\begin{array}{c} L_{real}=L_{pixel}\times r \end{array}$$

Where ${\text{RL}}_{\text{pixel}}$ represents the longest pixel span of the scale in the image, ${\text{RL}}_{\text{real}}$ denotes the actual physical length of the scale, r is the conversion factor between pixel length and real-world length, n is the total number of pixels along the skeleton, $\left({\text{x}}_{\text{i}},{\text{y}}_{\text{i}}\right)$ are the coordinates of the i-th pixel, ${\text{L}}_{\text{pixel}}$ is the pixel path length of the main stem, and $L_{\text{real}}$ is the actual physical length of the main stem.

## 3 Results

### 3.1 Evaluation metrics

This study employs mIoU, Recall, Precision, and F1-Score as evaluation metrics to assess the segmentation accuracy of the model. mIoU measures the overlap between the predicted segmentation and the ground truth segmentation, defined as Equation 17:

(17) $$\begin{array}{c} mIoU=\frac{TP}{TP+FP+FN}\times 100\% \end{array}$$

Recall reflects the proportion of correctly identified positive pixels out of all the actual positive pixels in the ground truth, and is defined as Equation 18:

(18) $$\begin{array}{c} Recall=\frac{TP}{TP+FN}\times 100\% \end{array}$$

Precision represents the proportion of true positive pixels among all the pixels predicted as positive by the model, and is defined as Equation 19:

(19) $$\begin{array}{c} Precision=\frac{TP}{TP+FP}\times 100\% \end{array}$$

The F1-score measures model performance on datasets with uneven class distribution Equation 20:

(20) $$\begin{array}{c} F1-Score=2\times \frac{Precision\times Recall}{Precision+Recall}\times 100\% \end{array}$$

Where TP denotes the number of pixels that are truly part of the main stem and correctly predicted by the model, FN refers to the number of pixels that are truly part of the main stem but incorrectly predicted as background, FP indicates the number of pixels that are truly background but incorrectly predicted as part of the main stem.

To assess the practicality of the model, we also report its number of parameters, computational complexity, and inference speed. The number of parameters refers to the total learnable parameters of the model. Computational complexity is quantified by the FLOPs needed for a single forward pass of the model. The model’s inference speed is expressed as frames processed per second (FPS), which indicates the maximum number of images the model can process per second under a fixed hardware setup.

### 3.2 Model training

All models were trained and evaluated under a unified software and hardware setup. The experimental setup comprised a workstation featuring an Intel Xeon Gold 6133 CPU, 128GB RAM, and an NVIDIA GeForce RTX 3090 GPU, running Python 3.7, PyTorch 1.12.1, and CUDA 11.3. All input images were uniformly scaled to a resolution of 512×512 pixels. The model training employed the Adam optimizer with an initial learning rate set to 0.0001 and a cosine annealing learning rate decay strategy. Training was performed with a batch size of 4 over 300 epochs. To leverage pre-trained knowledge and stabilize the training process, a staged training strategy was adopted. During the first 100 epochs, the parameters of the ResNet50 backbone network were frozen, with only the remaining parts of the model fine-tuned. This approach accelerated convergence and prevented the destruction of pre-trained weights in the early training stages. In the subsequent epochs, all parameters were unfrozen for full optimization and training. During training, the model’s performance was evaluated on the validation set every 10 epochs, and the best-performing model was saved.

**Figure 13:**
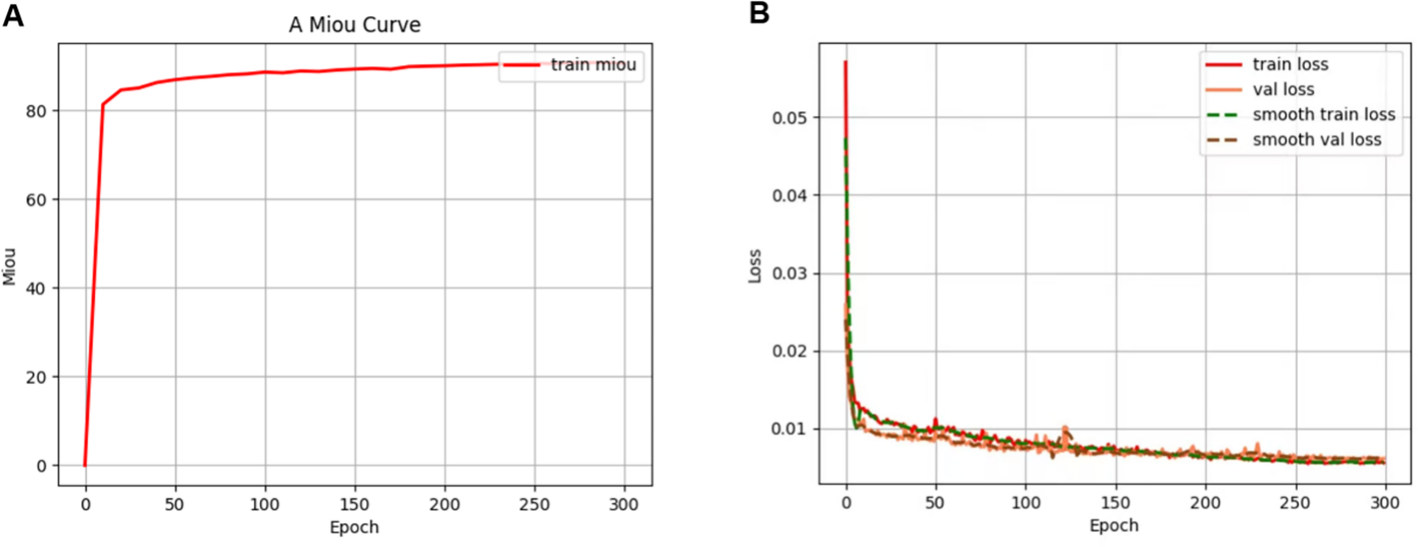
Training performance during optimization: **(A)** mIoU variation and **(B)** loss evolution.

The training process of the improved RAM-UNet model is dynamically illustrated in **Figure 13**. Thanks to the transfer learning from ImageNet pre-trained weights, the model showed rapid loss reduction during the initial training phases, demonstrating strong initialization characteristics. Both the training and validation loss curves smoothly decreased and ultimately stabilized at low values of approximately 0.05 and 0.06, respectively, with the two curves closely aligned throughout, showing no significant divergence. This indicates that the model was sufficiently optimized, with no overfitting, and exhibited good generalization performance. The mIoU on the validation set steadily increased with each training epoch, reaching saturation around the 250th epoch, ultimately achieving 90.58%. This confirms that the model’s capacity has been fully utilized, and the proposed improvements are effective. After 300 epochs of training, the model reached a stable and excellent state, with its convergence process providing a solid foundation for subsequent fair comparisons and performance analysis.

### 3.3 Segmentation performance comparison of different models

**Figure 14:**
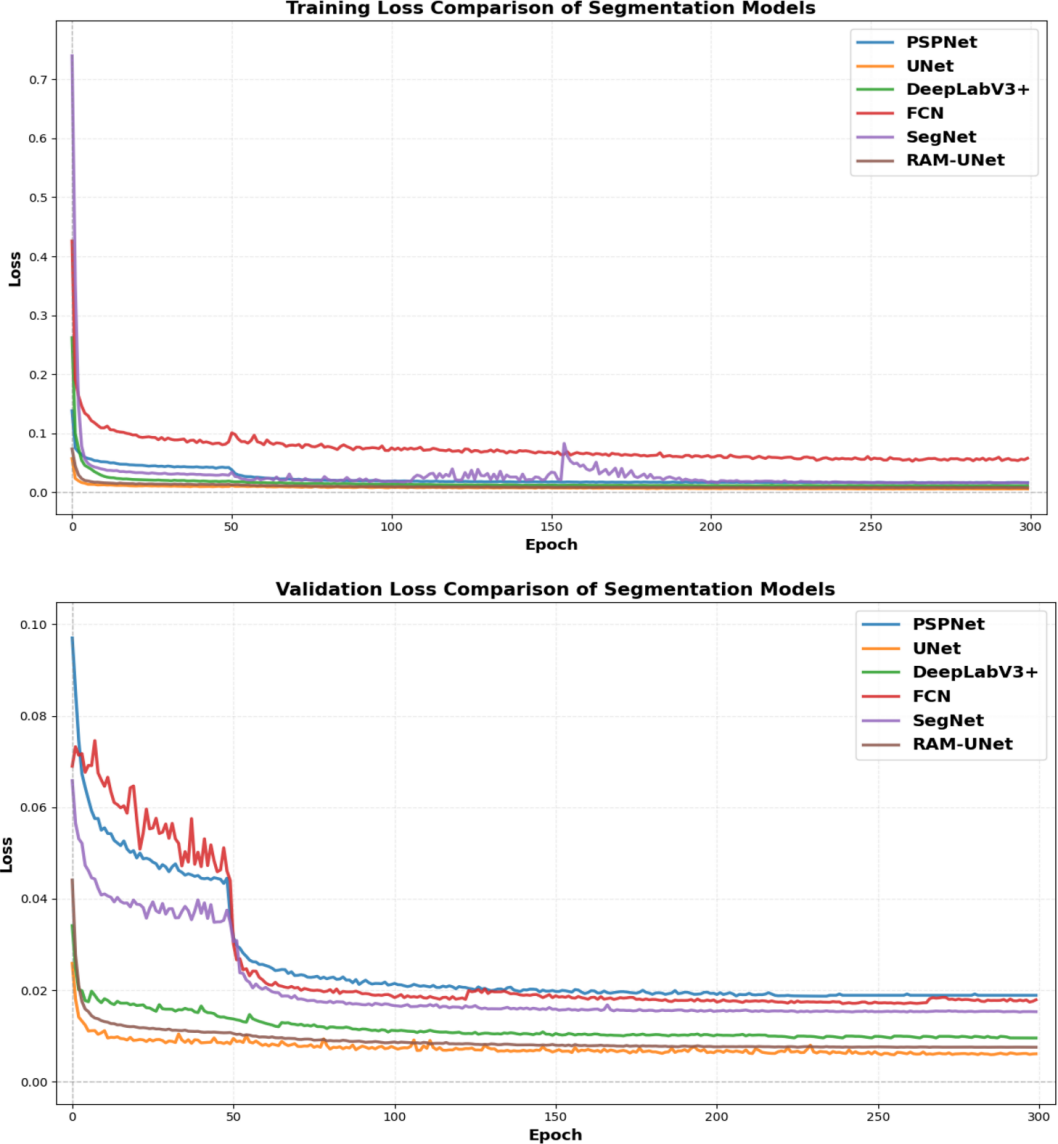
Comparative analysis of loss curves: **(A)** training loss and **(B)** validation loss.

A systematic evaluation of RAM-UNet was carried out on the soybean stem segmentation task, this study selected five representative semantic segmentation models—FCN, U-Net, DeepLabv3+, PSPNet, and SegNet—as baseline comparison models. All experiments were conducted under identical hardware and software conditions, using the same data preprocessing pipeline, data partitioning strategy, and training procedures, to ensure the objectivity and reproducibility of the comparison results. The convergence behavior during the training process reflects the stability and optimization efficiency of each model’s architecture. As shown in **Figure 14**, the training and validation loss curves of the compared models exhibit significant differences. Notably, the proposed RAM-UNet demonstrates the best optimization stability: its validation loss curve quickly converges and remains highly smooth from the mid-training stage, with almost no fluctuations. In contrast, the validation curves of models like the original U-Net show several noticeable increases in loss during the later stages of training. This indicates that RAM-UNet has a more robust optimization process and stronger generalization reliability, reducing performance uncertainty caused by training fluctuations.

As shown in **Table 1**, the RAM-UNet model attains an mIoU of 90.58%, improving by 6.41, 10.51, 22.41, and 17.37 percentage points over the original U-Net, DeepLabv3+, PSPNet, and SegNet, respectively. Additionally, it achieves the best recall (94.99%) and precision (94.58%) among all models. In terms of efficiency, RAM-UNet maintains an inference speed of 28.11 FPS with 47.08M parameters, which is comparable to the original U-Net (28.70 FPS). These results demonstrate that RAM-UNet not only significantly enhances segmentation accuracy but also achieves the optimal balance between accuracy and inference efficiency, providing an efficient and reliable solution for automated soybean stem phenotyping analysis.

**Table 1:** Segmentation results obtained by different methods.

Models	MIoU (%)	Recall (%)	Precision (%)	F1-Score (%)	FPS	FLOPs (G)	Params (M)
FCN	74.70	80.87	87.33	84.00	14.76	89.81	32.76
UNet	84.17	89.45	92.12	90.77	28.70	110.25	31.37
DeepLabv3+	80.07	84.10	90.86	87.34	12.72	166.85	54.71
PSPNet	68.17	72.13	83.29	77.29	21.49	118.45	46.72
SegNet	73.21	75.73	86.53	80.80	4.63	199.98	84.61
RAM-UNet	90.58	94.99	94.58	94.78n	28.11	184.89	47.08

**Figure 15:**
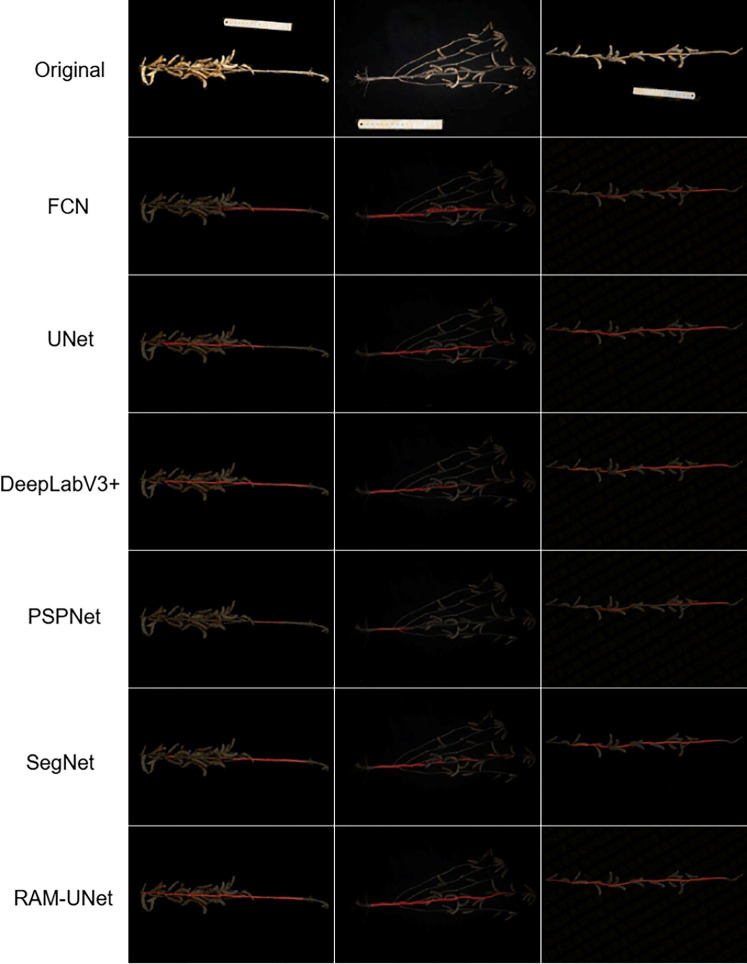
Segmentation results of various models.

**Figure 15** shows the comparison of segmentation results on the soybean stem dataset using different models. The performance of each model varies in terms of stem boundary recognition and pixel-level segmentation. When the soybean stem is naturally curved, partially occluded by pods, or when the features at the internode connections are ambiguous, the accuracy of traditional models significantly decreases. Models such as PSPNet, FCN, and DeepLabv3+ show issues like stem breakage, rough edges, and misidentification of adjacent background regions. Although U-Net maintains relatively good continuity, its ability to adapt to curved stem shapes is limited, with noticeable loss of details. In contrast, the proposed RAM-UNet model in this study effectively addresses these challenges by introducing deformable convolutions to adaptively capture the curvature and deformation features of the stem. The ASPP module integrates multi-scale information to enhance segmentation consistency for stems with varying thicknesses. The CBAM attention mechanism focuses on the main stem area, reducing background interference. Finally, the MSAA module optimizes feature fusion, ensuring accurate recovery of edge contours. Experimental results demonstrate that the proposed model maintains stem segmentation continuity and integrity even in complex backgrounds, providing reliable support for the accurate extraction of soybean phenotypic parameters.

### 3.4 Experiments with different backbones

An investigation into the effects of various backbone networks on soybean stem segmentation performance was performed, we compared VGG16, ResNet50, ResNet101, and the proposed improved ResNet50-DCN within the basic U-Net framework. The experimental results are shown in **Table 2**. Compared to VGG16, deeper networks such as ResNet50 and ResNet101 showed improvements in mIoU by approximately 1.0 and 0.9 percentage points, respectively. However, the gain from ResNet101 over ResNet50 was minimal, and the computational cost increased significantly, indicating that merely increasing network depth provides diminishing returns for soybean stem segmentation. ResNet50-DCN, featuring deformable convolution, outperformed the other backbone networks, attaining an mIoU of 86.38%. This model only added about 2.4M parameters and 6.5 GFLOPs over ResNet50, yet it improved the mIoU by 0.53 percentage points. This result strongly supports that deformable convolution, through adaptive deformation modeling, can more effectively capture the curved shape of the stem. The performance improvement it brought was far greater than the marginal gains from simply increasing network depth, thus validating the necessity and superiority of the backbone network improvement.

**Table 2:** Results of different backbone experiments.

Backbone network	mIoU (%)	Recall (%)	Precision (%)	F1-Score (%)	FLOPs (G)	Params (M)
VGG16	84.82	89.88	92.34	91.10	451.71	138.00
ResNet50	85.85	90.92	92.78	91.84	152.38	39.21
ResNet101	85.72	90.65	92.85	91.76	215.47	58.97
ResNet50-DCN	86.38	91.62	93.12	92.38	158.92	41.56

### 3.5 Experiments with different loss functions

To address the class imbalance issue caused by sparse foreground pixels, this study compared the performance of cross-entropy loss, Dice loss, and their weighted composite loss, according to **Table 3**. Compared to cross-entropy loss, the Dice loss, which is optimized for region overlap, improved the mIoU by 0.9 percentage points, validating its effectiveness in enhancing foreground pixel recall. The composite loss function integrating cross-entropy and Dice produced the top performance, attaining an mIoU of 87.45% and the highest F1-score. This demonstrates that the composite loss effectively combines the stable pixel-level classification ability of cross-entropy with the structural optimization capability of Dice loss, making it a superior choice for addressing the class imbalance issue in this task.

**Table 3:** Results of different loss function experiments.

Loss Functions	mIoU (%)	Recall (%)	Precision (%)	F1-Score (%)
Cross Entropy loss (CE)	86.38	91.62	93.12	92.38
Dice loss	87.28	92.18	93.45	92.81
CE + Dice	87.45	92.32	93.58	92.94

### 3.6 Component-wise ablation analysis

#### 3.6.1 Ablation study on the C-ASPP structure

A systematic evaluation of the C-ASPP module design was conducted, this section systematically evaluates the contribution of different dilation rate configurations and the CBAM attention mechanism to segmentation performance, using the ResNet50-DCN backbone network as the baseline (M0). A total of nine model configurations were designed, and their specific composition and performance are shown in **Table 4**.

**Table 4:** Ablation study results of C-ASPP module.

Module	Setup Instructions	mIoU (%)	Recall (%)	Precision (%)	F1-Score
M0	Baseline	87.45	92.32	93.58	92.94
M1	+ASPP (6, 12, 18)	87.96	91.23	92.58	93.10
M2	+ASPP (2, 7, 15)	87.88	91.18	92.55	93.05
M3	+ASPP (1, 2, 4, 8)	87.85	91.15	92.48	92.98
M4	+ASPP (1, 3, 6, 12)	88.12	91.78	92.89	93.25
M5	+ASPP (1, 2, 7, 15)	88.43	92.14	93.18	93.55
M6	+ASPP (1, 2, 7, 15) + CAM	88.72	92.38	93.42	93.70
M7	+ASPP (1, 2, 7, 15) + SAM	88.95	92.62	93.67	93.90
M8	+ASPP (1, 2, 7, 15) + CBAM	89.18	92.89	93.85	94.12

1. M1-M5: These models are based on M0, with different dilation rate configurations of the ASPP module added to investigate the impact of dilation rate combinations on multi-scale perception.
2. M6-M8: These models are based on the optimal dilation rate configuration from M5, adding Channel Attention Mechanism (CAM), Spatial Attention Mechanism (SAM), and their combination, CBAM, to evaluate the enhancement effect of the attention mechanism.

Based on the ablation study results in **Table 4**, the following conclusions can be drawn: Comparing M1 (standard ASPP), M4 (linear dilation rates), and M5 (proposed configuration), it is evident that M5 achieves the best performance across all metrics. This demonstrates that the dilation rate combination specifically designed for soybean plant scale (1, 2, 7, 15) can more effectively aggregate multi-scale contextual information. Notably, M3, which suffers from a grid effect due to common factors in the dilation rates, shows the lowest performance, thereby reinforcing the necessity of the dilation rate design proposed in this study. Comparing M2 (without r=1 branch) and M5 (with r=1 branch), the absence of the r=1 branch leads to a decrease in model performance. This demonstrates that the r=1 branch, which extracts the most basic local features, is essential for maintaining the fine details of the stem edges and ensuring segmentation continuity. Adding Channel Attention (M6) and Spatial Attention (M7) to M5 results in stable improvements in performance. The integration of both attention mechanisms into CBAM (M8) achieves the highest accuracy among all models. This shows that channel attention enhances the model’s response to the main stem semantics, while spatial attention focuses on high-detail regions such as edges. The synergy of both mechanisms comprehensively enhances the model’s feature selection capability.

#### 3.6.2 Evaluation of different modules via ablation

The influence of the introduced architectural refinements on the segmentation quality of the UNet network was systematically evaluated, ablation experiments were conducted on a self-built dataset. Using the UNet model as the baseline, five groups of ablation experiments were designed, and semantic segmentation evaluation metrics were employed. The experimental results are presented in **Table 3**, where “√” indicates that the specified module was used.

1. G0: The original U-Net architecture was used as a baseline for performance and complexity comparison.
2. G1: The encoder was replaced with ResNet50-DCN, enabling the network to dynamically adapt its receptive field for more effective modeling of the curved morphology of soybean stems.
3. G2: By integrating the C-ASPP module at the encoder’s end in G1, the model better aggregates and selects information across multiple scales.
4. G3: The MSAA module was added to the decoder of G1 to optimize the feature fusion quality in the decoding path, which directly and effectively improves the detail and continuity of the segmentation results.
5. G4: By combining ResNet50-DCN, C-ASPP, and MSAA, G4 represents the complete proposed RAM-UNet model.

As shown in **Table 5**, substituting the original U-Net encoder with a deformable ResNet50 backbone led to an additional 10.19M parameters, with the mIoU improving by 3.28 percentage points to 87.45%. Building on this, introducing the C-ASPP module added an additional 3.73M parameters, contributing a further 2.87 percentage points increase in mIoU, validating the effectiveness of multi-scale context enhancement. The inclusion of the MSAA module, with an additional 5.87M parameters, achieved a 2.40 percentage point performance improvement, demonstrating the importance of optimizing feature fusion in the decoder. Ultimately, the complete model integrating all improvements achieved a mIoU of 90.58%, a 6.41 percentage point improvement over the original baseline, with a recall rate of 94.99%, striking an excellent balance between accuracy and efficiency.

**Table 5:** Ablation results of individual module.

Model Stage	Resnet50-DCN	C-ASPP	MSAA	mIoU (%)	Recall (%)	Precision (%)	F1-Score (%)	Params (M)
G0				84.17	89.45	92.12	90.77	31.37
G1	✓			87.45	92.32	93.58	92.94	41.56
G2	✓	✓		90.32	93.85	94.45	94.15	45.29
G3	✓		✓	89.85	93.25	94.12	93.68	47.43
G4	✓	✓	✓	90.58	94.99	94.58	94.78	48.84

### 3.7 Testing on the PASCAL VOC 2012

**Figure 16:**
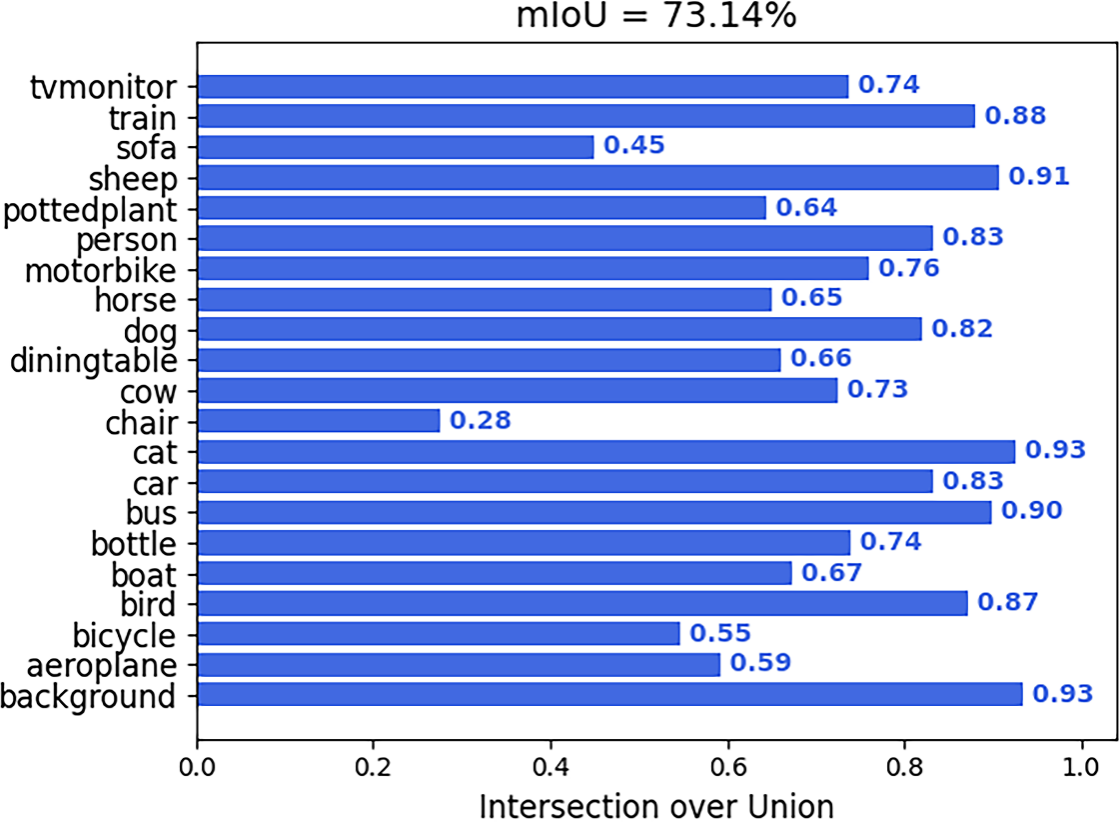
Category-wise mIoU scores.

To evaluate the model’s cross-domain performance, experiments were conducted on the well-known Pascal VOC 2012 dataset (Everingham et al., 2010). It provides 20 object categories and diverse image content, which fundamentally differs from the agricultural images in terms of semantics, textures, and context. This presents a challenging benchmark for verifying the robustness of the model’s core feature extractor. **Figure 16** presents results on the Pascal VOC 2012 dataset, illustrating the model’s effective foreground extraction for unseen categories. To objectively assess its performance, we compared our model with several classical semantic segmentation models that were specifically trained on this dataset. **Table 6** presents the mIoU results. Our model yielded an mIoU of 73.14%, outperforming some of the multi-class segmentation models that were specifically trained on this dataset in several categories. This strong result demonstrates that by employing ResNet50-DCN as the backbone and innovatively integrating the C-ASPP module to aggregate multi-scale context and the MSAA module for refined feature fusion, the model learns a universal visual representation that is highly sensitive to object structures and edges. While there is still a reasonable gap between the overall performance and the state-of-the-art fully-supervised models, the superior performance on several specific categories robustly validates the significant advantages and practical potential of the proposed architecture in terms of feature learning and generalization across different domains.

**Table 6:** Performance comparison of different segmentation models.

Classification	FCN	UNet	DeepLabv3+	PSPNet	SegNet	RAM-UNet
aeroplane	0.77	0.79	0.84	0.82	0.75	0.59
bicycle	0.34	0.52	0.67	0.62	0.31	0.55
bird	0.69	0.73	0.81	0.78	0.61	0.87
boat	0.50	0.61	0.71	0.68	0.51	0.67
bottle	0.60	0.65	0.72	0.70	0.49	0.74
bus	0.75	0.81	0.87	0.85	0.76	0.90
car	0.74	0.78	0.83	0.81	0.64	0.83
cat	0.78	0.80	0.86	0.84	0.69	0.93
chair	0.21	0.31	0.42	0.38	0.24	0.28
cow	0.62	0.68	0.77	0.74	0.61	0.73
diningtable	0.47	0.55	0.64	0.61	0.55	0.66
dog	0.72	0.76	0.82	0.80	0.62	0.82
horse	0.63	0.70	0.78	0.76	0.66	0.62
motorbike	0.76	0.79	0.85	0.83	0.70	0.76
person	0.74	0.77	0.82	0.80	0.74	0.83
plant	0.45	0.52	0.60	0.58	0.37	0.64
sheep	0.72	0.76	0.83	0.81	0.64	0.91
sofa	0.37	0.45	0.54	0.51	0.41	0.45
train	0.71	0.78	0.84	0.82	0.68	0.88
tvmonitor	0.55	0.68	0.74	0.72	0.53	0.74
mIoU	0.62	0.67	0.74	0.72	0.59	0.73

### 3.8 Evaluation of soybean main stem length calculation results

**Figure 17:**
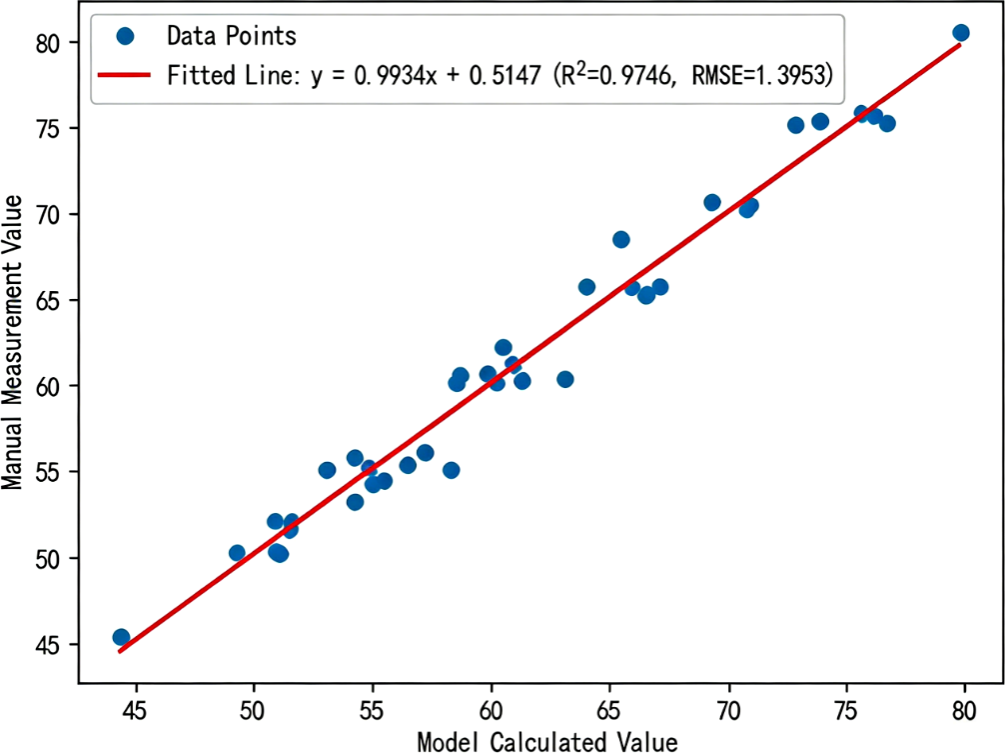
Correlation between automated stem length measurement and manual measurement.

To validate the reliability and practicality of the RAM-UNet model’s segmentation results in real-world applications, this study randomly selected mature soybean plants from the validation set. The RAM-UNet model was used to perform stem segmentation, followed by skeletonization and morphological closing operations on the output masks to achieve automated calculation of the main stem length. The results were evaluated in terms of both accuracy and efficiency. The model’s computed results were compared with manually measured values, demonstrating the reliability of the method for practical use. As shown in **Figure 17**, the linear regression analysis yielded an R² value of 0.9746, and the RMSE was 1.3953 cm. The average processing time for the entire pipeline was only 1.85 seconds per plant, indicating that the proposed method offers good feasibility and precision.

## 4 Discussion

Semantic segmentation techniques have become an essential tool for agricultural phenotyping analysis. General architectures such as U-Net and DeepLabv3+ have demonstrated strong performance in the segmentation of various crop organs. However, the mature soybean plant’s main stem and pods have highly similar color textures and severe occlusions, and the stem itself exhibits continuous scale variations from the base to the tip. These challenges result in limited segmentation accuracy for general models in such complex scenarios, leading to issues like segmentation breaks and misclassification. RAM-UNet is proposed to tackle these challenges. This model uses U-Net as a baseline and incorporates a ResNet50 backbone in the encoder to enhance feature representation. The core convolutions are replaced with deformable convolutions, enabling the network to adaptively adjust its receptive field and accurately capture the curvature and deformation of the stem. At the end of the encoder, an improved C-ASPP module is introduced, with gradientized dilation rates (1, 2, 7, 15) to match the stem’s morphological changes. Additionally, the CBAM attention mechanism is embedded to effectively aggregate key information from local details to global layouts, enhancing the model’s discriminative ability for similar organs. In the decoder, the MSAA module is incorporated to recalibrate the skip connection features using multi-scale attention, strengthening key details and suppressing noise introduced by occlusions, thus optimizing the recovery of contours under complex occlusions. To handle the class imbalance resulting from sparse foreground pixels, the model employs a combined Dice and cross-entropy loss.

Although the combination of deformable convolutions, attention mechanisms, and multi-scale feature aggregation has been individually reported in the literature, this study is the first to integrate them synergistically into the U-Net encoder-decoder architecture. The model was specifically optimized for the unique challenges of mature soybean stems, which exhibit highly similar colors and textures, significant curvature, and severe occlusion. On the custom-built dataset of mature soybean plant stem segmentation, RAM-UNet achieved an mIoU of 90.58%, with recall and precision rates of 94.99% and 94.58%, respectively. Its overall segmentation performance significantly outperforms popular models such as U-Net, DeepLabv3+, PSPNet, and SegNet. Despite an increase in model parameters, it maintained good inference efficiency at 28.11 FPS. The automated stem length measurement based on segmentation results showed a high correlation with manual measurements (R² = 0.9746), validating its practical applicability. The model also demonstrated good generalization performance on the PASCAL VOC 2012 dataset, achieving an mIoU of 73.14%, further proving the universality of the learned features.

This study, however, is subject to certain shortcomings. First, model training and validation were primarily based on images collected under controlled laboratory conditions, with uniform lighting and simple backgrounds. The model has not yet been fully validated and adapted for more complex field environments. Second, although the current dataset covers the main morphological variations, it may not adequately address the local color and texture degradation of the main stem caused by extreme diseases, pests, or mechanical damage. This limitation could affect the model’s segmentation stability under such abnormal conditions. Future work will focus on constructing a large-scale main stem segmentation dataset that includes multiple environments, cultivars, and growth stages. Additionally, we aim to explore enhanced segmentation methods integrating multimodal information and investigate lightweight models and edge deployment strategies to promote the widespread application of this technology in practical agricultural production systems and field-based phenotyping platforms.

## 5 Conclusion and outlook

This study addresses the critical need for automated and precise segmentation of the main stem in mature soybean plant phenotyping. To tackle the issues of poor adaptation to curved shapes and insufficient continuity in occluded regions, commonly observed in existing models, we propose an improved segmentation network called RAM-UNet. On our custom dataset, RAM-UNet achieved a remarkable mIoU of 90.58%, significantly outperforming mainstream models. The model generates continuous and accurate main stem segmentation masks, and based on the segmentation results, it enables highly accurate main stem length measurement, providing a reliable technical foundation for soybean phenotypic parameter analysis.

Future research will focus on validating the model’s generalization ability across different environmental conditions and soybean varieties, and exploring the extension of this algorithmic framework to segmentation tasks for other key crop organs, such as maize stems and wheat spikes. Future applications will transition from laboratory settings to field-based high-throughput phenotyping platforms, providing broader technical support for precision breeding and crop management in smart agriculture.

## Data Availability

The raw data supporting the conclusions of this article will be made available by the authors, without undue reservation.
